# Effects of Traditional Chinese Mind–Body Exercise on Physical Function in Older Adults with Sarcopenia and Frailty: A Meta-Analysis of Randomized Controlled Trials

**DOI:** 10.3390/healthcare14162664

**Published:** 2026-08-21

**Authors:** Jingtao Zhou, Wi-Young So, Minhye Shin

**Affiliations:** 1 Department of Sports Guidance, Kyungil University, Gyeongsan 38428, Republic of Korea; 20259288@kiu.kr; 2 Department of Sports Medicine, College of Humanities, Korea National University of Transportation, Chungju 27469, Republic of Korea; wowso@ut.ac.kr

**Keywords:** frailty, Health Qigong, meta-analysis, older adults, sarcopenia, Tai Chi, traditional Chinese mind–body exercise

## Abstract

**Background and Objectives:** Sarcopenia and frailty are associated with falls, disability, and functional decline in older adults. This review evaluated traditional Chinese mind–body exercise (TCMBE), including Tai Chi and Health Qigong, in adults aged 60 years or older with clinically defined sarcopenia or frailty. **Materials and Methods:** Randomized controlled trials identified through December 2024 from six English- and Chinese-language databases were synthesized. The analyses included physical performance and secondary outcomes and explored sarcopenia and frailty as study-level subgroups. **Results:** Twenty-two trials involving 1660 participants were included. TCMBE was associated with a shorter Timed Up and Go Test time (mean difference [MD], −1.17 s; 95% CI, −1.80 to −0.54; *p* < 0.01; I^2^ = 74%), a higher Short Physical Performance Battery score (MD, 0.99 points; 95% CI, 0.58 to 1.40; *p* < 0.01; I^2^ = 24%), and greater grip strength (MD, 2.02 kg; 95% CI, 0.58 to 3.47; *p* = 0.006; I^2^ = 89%). The 6 min walk test estimate was borderline and imprecise (MD, 11.76 m; 95% CI, 0.10 to 23.43; *p* = 0.05; I^2^ = 0%), and no clear overall effects were observed for gait speed, the 30 s chair stand test, sit-to-stand time, Berg Balance Scale score, muscle mass, depressive symptoms, or quality of life. Several analyses showed substantial heterogeneity, and some subgroup estimates were based on only one or two studies. **Conclusions:** Low-certainty evidence suggests that TCMBE may produce small improvements in TUGT performance, SPPB scores, and grip strength. Very-low-certainty evidence leaves its effects on gait speed and quality of life uncertain. Risk-of-bias concerns, heterogeneity, imprecision, and uncertain clinical significance limit confidence in the current estimates. The available evidence is insufficient to support routine implementation, and larger, rigorously conducted randomized controlled trials are needed.

## 1. Introduction

Age-related health challenges are becoming increasingly prominent with the rapid growth of the aging population. Aging, an inevitable biological process, is characterized by the progressive accumulation of damage and functional decline at the molecular, cellular, and systemic levels. This gradual deterioration significantly compromises the body’s ability to adapt to internal and external stressors, resulting in a marked reduction in physiological reserve. Consequently, aging substantially increases susceptibility to chronic diseases. Among the many health conditions associated with aging, sarcopenia and frailty are two of the most prevalent and closely related syndromes. Together, they contribute substantially to functional decline, reduced quality of life, disability, and mortality [1].

Sarcopenia and frailty are two common geriatric syndromes with closely related pathophysiological mechanisms and clinical manifestations [2,3]. The most prominent clinical feature of both syndromes is impaired physical function, which can lead to adverse health outcomes such as falls, disability, and fractures, thereby severely compromising the quality of life of older adults [4]. Current therapeutic interventions primarily include pharmacological treatments and nutritional supplementation; however, these approaches have several limitations. Pharmacological therapies are often associated with adverse effects and potential risks.

In contrast, exercise interventions contribute to the prevention and delay of sarcopenia and frailty. Among the common exercise modalities, resistance training, aerobic exercise, and flexibility training effectively improve muscle mass and physical function [5,6]. However, these conventional exercise modalities impose relatively high physical demands, often require specialized equipment or facilities, and carry a risk of injury or excessive fatigue, which may hinder long-term adherence among older adults. In particular, traditional Chinese mind–body exercises (TCMBEs), including Tai Chi and Health Qigong practices such as Baduanjin, serve as promising alternatives.

Health Qigong comprises low-intensity, easy-to-perform exercises and represents an integrated mind–body exercise system that promotes biomechanical optimization, neuromuscular regulation, and improved energy metabolism [7,8]. From a mechanistic perspective, Health Qigong offers several advantages over conventional forms of exercise. It integrates deep, slow, rhythmic abdominal breathing with gentle, flowing movements. This movement-breath integration enhances vagal tone and modulates autonomic nervous system balance by shifting the body from a stress-dominated state (increased sympathetic activity) to a state of relaxation and recovery (increased parasympathetic activity) [9]. This autonomic modulation may help reduce resting heart rate and blood pressure, alleviate anxiety, and improve systemic inflammation and metabolic status through the brain–gut-muscle axis [10].

Randomized trials suggest that mind–body exercise may improve physical function in older adults; however, the evidence base remains fragmented. A previous meta-analysis evaluated Tai Chi in older adults with sarcopenia or frailty, but it did not encompass other commonly used traditional Chinese mind–body exercises, such as Baduanjin, Yijinjing, and Wuqinxi, and it preceded several recent trials [11]. In addition, uncertainty remains regarding whether effects are consistent across the clinically distinct populations of sarcopenia and frailty and across mobility, muscle strength, balance, body-composition, psychological, and activities-of-daily-living outcomes.

Accordingly, this meta-analysis aimed to update and broaden the evidence by synthesizing randomized controlled trials of prespecified traditional Chinese mind–body exercises in adults aged 60 years or older with clinically defined sarcopenia or frailty. The primary objective was to estimate pooled effects on physical-performance outcomes. Secondary objectives were to evaluate muscle, psychological, cognitive, quality-of-life, and activities-of-daily-living outcomes; examine sarcopenia and frailty as study-level subgroups; and explore potential sources of between-study heterogeneity.

## 2. Materials and Methods

This meta-analysis was reported in accordance with the Preferred Reporting Items for Systematic Reviews and Meta-Analyses (PRISMA) 2020 statement. A completed PRISMA 2020 checklist is provided as Table S1. The review was retrospectively registered in PROSPERO (CRD42025633826; registration date: 15 April 2025). Registration was completed after the database search had commenced because preparation and administrative submission of the registration record were delayed. However, the eligibility criteria and outcomes were specified before quantitative data synthesis, and no substantive methodological changes were made after registration.

### 2.1. Search Strategy

A systematic search was conducted across the following databases: PubMed, Web of Science, Embase, the Cochrane Library, China National Knowledge Infrastructure (CNKI), and the VIP Database. Specifically, the search was conducted for randomized controlled trials (RCTs) published between 1989 and December 2024. Controlled vocabulary was combined with free-text terms, including appropriate Medical Subject Headings (MeSH) in PubMed and Emtree terms in Embase. The intervention terms included Tai Chi, Tai Ji, Qigong, Health Qigong, Baduanjin, Yijinjing, Wuqinxi, and Liuzijue, together with relevant spelling variants. The Chinese search terms included “衰弱,” “虚弱,” “肌少症,” “少肌症,” “肌肉减少症,” “太极,” “太极拳,” “气功,” “健身气功,” “八段锦,” “易筋经,” “五禽戏,” “六字诀,” “随机,” and “随机对照试验.” Database-specific field tags, truncation, proximity operators, and validated randomized trial filters were used where applicable. No additional randomized trial filter was applied to the CENTRAL Trials collection. The reference lists of the included studies were also screened to identify additional relevant publications. After duplicate records were removed, the studies were screened based on their titles and abstracts to exclude those that did not meet the inclusion criteria. Two reviewers (JZ and MS) independently assessed the eligibility of the studies, and only studies with available full-text articles were included. Any disagreements were resolved through discussion with the third author.

### 2.2. Inclusion and Exclusion Criteria

Studies were eligible if they: (1) were individually randomized or cluster-randomized controlled trials with an accessible full-text report; (2) enrolled adults aged ≥60 years with sarcopenia, frailty, prefrailty, or cognitive frailty diagnosed using an explicitly reported criterion or guideline; (3) evaluated a prespecified traditional Chinese mind–body exercise—Tai Chi, Baduanjin, Yijinjing, Wuqinxi, or another eligible Health Qigong form—either alone or as an adjunct to a co-intervention applied equally across groups; (4) included an inactive, usual-care, wait-list, health-education, or active-exercise comparator; and (5) reported at least one prespecified eligible outcome, irrespective of whether the result was reported in a form suitable for quantitative synthesis.

Studies were excluded if they: (1) were non-randomized, non-original, or duplicate publications; (2) enrolled participants younger than 60 years without separately extractable data for eligible older adults; (3) did not report an eligible sarcopenia or frailty definition; (4) did not evaluate an eligible traditional Chinese mind–body exercise or comparator; (5) did not report any prespecified eligible outcome; or (6) had no accessible full-text report.

Eligibility for the systematic review was determined independently of the availability of analyzable numerical data. A study meeting the systematic-review eligibility criteria was included in a particular outcome-specific meta-analysis only when it reported the relevant outcome and provided, or allowed calculation of, the data required to estimate an effect size and its variance. A study was not excluded from the systematic review solely because its eligible outcome data were insufficient for quantitative synthesis. When multiple reports described the same trial, they were linked and treated as one study (Table 1).

### 2.3. Data Extraction

Data from the included studies were independently extracted by two reviewers, and any discrepancies were resolved through discussion until consensus was reached among all authors. The following information was collected from each study: the first author’s name, year of publication, whether participants were diagnosed with frailty or sarcopenia, sample size, age, sex, descriptions of the intervention and control conditions, outcome measures, and other data relevant to geriatric syndromes.

### 2.4. Risk of Bias Assessment

Two reviewers (JZ and MS) independently assessed the risk of bias in individual trial results using the revised Cochrane Risk of Bias 2 tool (RoB 2). The following five RoB 2 domains were assessed: (1) bias arising from the randomization process; (2) bias due to deviations from intended interventions; (3) bias due to missing outcome data; (4) bias in measurement of the outcome; and (5) bias in selection of the reported result. The RoB 2 signaling questions and algorithms were used to assign domain-level and overall judgments of low risk of bias, some concerns, or high risk of bias for each specific result. Overall judgments were not interpreted as measures of general study quality. Disagreements were resolved through discussion and, when necessary, adjudication by a third reviewer.

Two reviewers independently assessed the certainty of evidence for the five critical outcomes included in the Summary of Findings table: gait speed, Timed Up and Go Test, Short Physical Performance Battery, grip strength, and quality of life. The Grading of Recommendations Assessment, Development and Evaluation (GRADE) approach was used. Evidence from randomized controlled trials initially received a high-certainty rating and was evaluated for potential downgrading according to five domains: risk of bias, inconsistency, indirectness, imprecision, and publication bias. Each domain was classified as not serious, serious, or very serious, as applicable. The final certainty of evidence was categorized as high, moderate, low, or very low. Disagreements were resolved through discussion and, when necessary, consultation with a third reviewer.

### 2.5. Statistical Analysis

A meta-analysis was performed using Stata version 15.0 (StataCorp LP, College Station, TX, USA). For continuous outcomes, mean differences (MDs) or standardized mean differences (SMDs) were calculated with 95% confidence intervals (CIs, depending on the consistency of the measurement scales across studies. Heterogeneity among studies was assessed using the Chi-square test and quantified using the I^2^ statistic. An I^2^ value of <50% was considered to indicate low heterogeneity, 50–75% moderate heterogeneity, and >75% high heterogeneity. A fixed-effect model was applied when heterogeneity was low (I^2^ < 50% and *p* > 0.10). Otherwise, a random-effects model was used. Subgroup analyses were conducted according to the type of condition (sarcopenia versus frailty). Finally, publication bias was assessed using funnel plots and Egger’s regression test when 10 or more studies were included in the meta-analysis for a specific outcome. Statistical significance was set at *p* < 0.05.

## 3. Results

### 3.1. Literature Search

**Figure 1:**
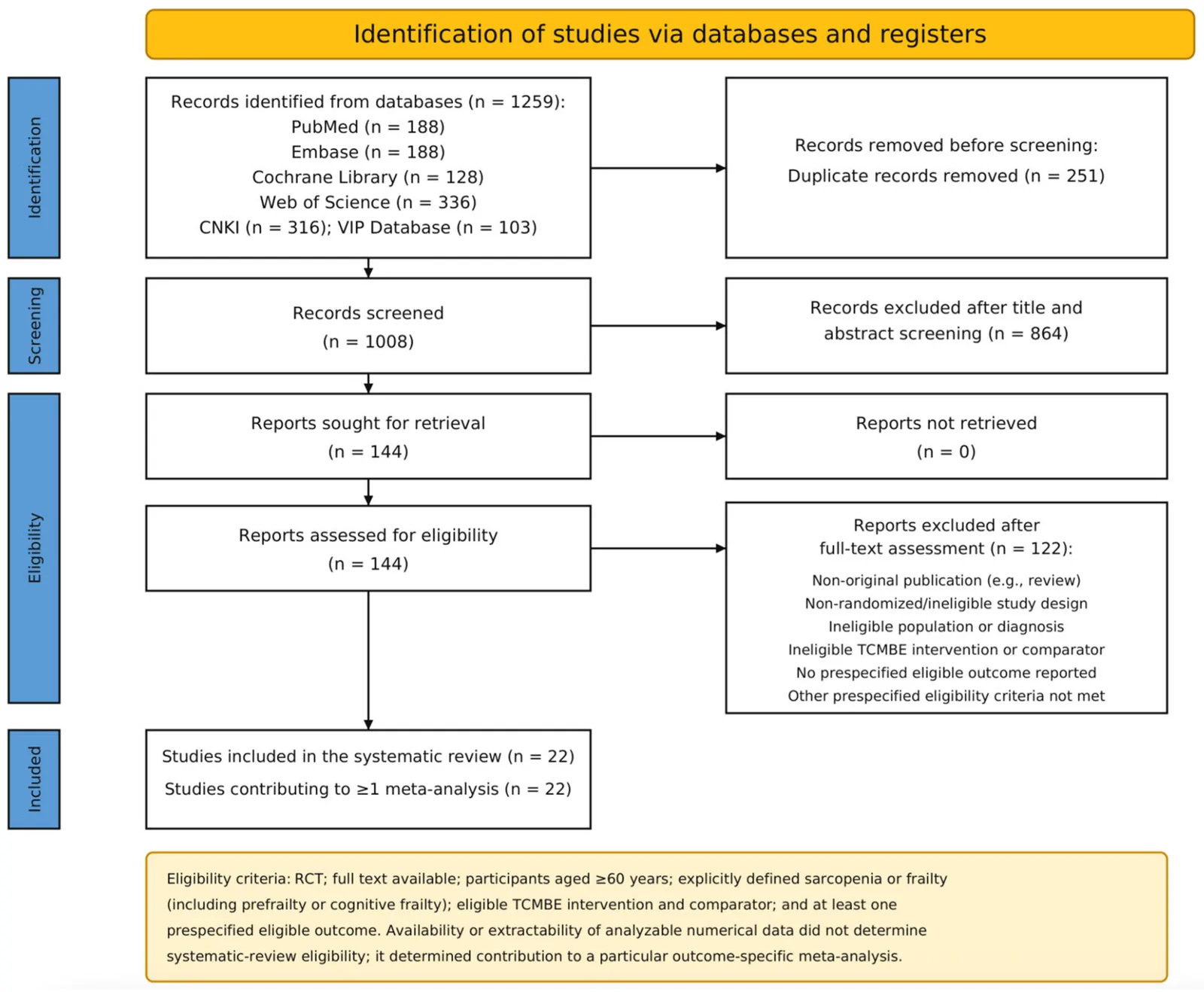
Preferred Reporting Items for Systematic Reviews and Meta-Analyses (PRISMA) flow diagram.

Following full-text assessment, 122 reports were excluded for the prespecified reasons presented in Figure 1, including non-original publications, ineligible study designs, ineligible populations or diagnoses, ineligible interventions or comparators, and failure to report any prespecified eligible outcome. Twenty-two RCTs met the eligibility criteria and were included in the systematic review. The availability of analyzable numerical data did not determine eligibility for the systematic review but was assessed separately for each outcome-specific meta-analysis. All 22 included studies provided sufficient data for at least one outcome and therefore contributed to at least one meta-analysis; however, the number of contributing studies varied across the individual outcome-specific meta-analyses. The study-selection process is presented in the PRISMA flow diagram (Figure 1).

### 3.2. Basic Characteristics of the Included Studies

The 22 RCTs involved a total of 1660 participants, including 1059 with frailty and 601 with sarcopenia. Of these, 834 participants were assigned to the intervention groups, and 826 were assigned to the control groups. The basic characteristics of the included studies are summarized in Table 2. The studies were published between 2006 and 2024, with sample sizes ranging from 21 to 311 participants. The experimental interventions included Health Qigong practices, such as Baduanjin, Tai Chi, and Yijinjing, administered either alone or in combination with other exercise modalities (e.g., resistance training and combined strength and endurance training). The control groups received various interventions, including wellness education, cognition–action exercise programs, usual care, and health education.

### 3.3. Risk of Bias in the Included Studies

**Figure 2:**
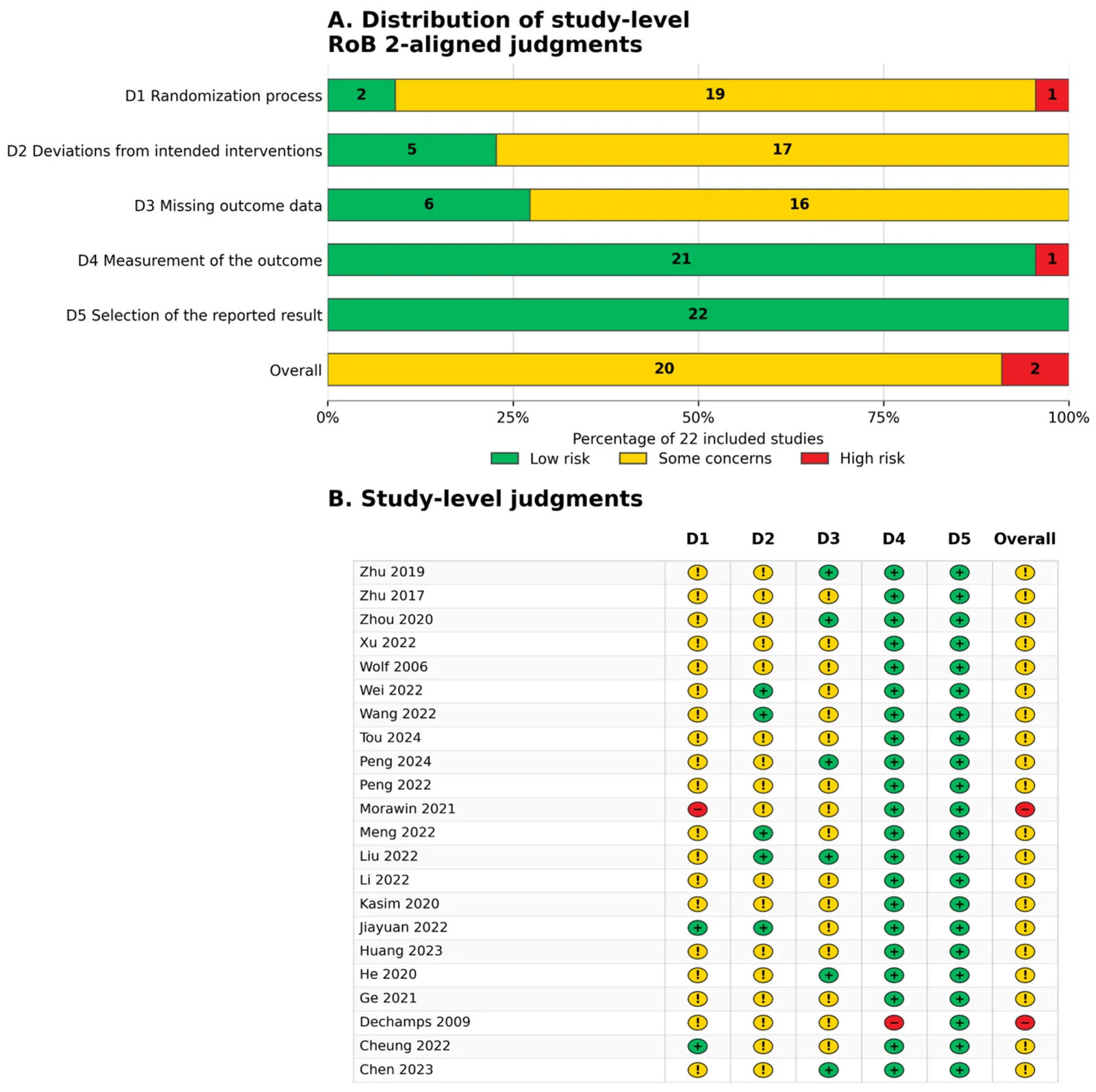
Risk-of-bias assessment of the included studies using the Cochrane Risk of Bias 2 (RoB 2) tool. (**A**) Distribution of study-level judgments across the 22 included studies. Values within the bars indicate the number of studies. (**B**) Individual judgments for each study. D1, randomization process; D2, deviations from intended interventions; D3, missing outcome data; D4, measurement of the outcome; D5, selection of the reported result. Green indicates low risk, yellow indicates some concerns, and red indicates high risk. The included studies are reported in References [12,13,14,15,16,17,18,19,20,21,22,23,24,25,26,27,28,29,30,31,32,33].

Figure 2 presents a study-level summary of the risk-of-bias judgments for the 22 included randomized controlled trials using the five RoB 2-aligned domains.

For bias arising from the randomization process (D1), two studies were judged to be at low risk of bias, 19 raised some concerns, and one was judged to be at high risk of bias. The concerns were primarily related to insufficient reporting of random-sequence generation and allocation concealment. For bias due to deviations from intended interventions (D2), five studies were judged to be at low risk of bias, whereas 17 raised some concerns because information regarding participant or personnel awareness of intervention assignment and deviations from the intended interventions was incomplete. For bias due to missing outcome data (D3), six studies were judged to be at low risk of bias and 16 raised some concerns because of participant loss to follow-up, missing data, or insufficient reporting of how missing data were addressed.

For bias in measurement of the outcome (D4), 21 studies were judged to be at low risk of bias and one study was judged to be at high risk of bias. All 22 studies were provisionally judged to be at low risk for bias in selection of the reported result (D5), based on the reporting information available in the included articles.

The completed GRADE assessment is presented in Table A1. The certainty of evidence was rated as low for TUGT, SPPB, and grip strength and very low for gait speed and quality of life. The certainty ratings were reduced primarily because of risk-of-bias concerns, substantial or considerable between-study heterogeneity, and imprecision. Accordingly, the statistically significant pooled estimates for TUGT, SPPB, and grip strength should be interpreted as possible rather than definitive intervention effects, whereas the effects on gait speed and quality of life remain highly uncertain.

### 3.4. Meta-Analysis of the Effects of Health Qigong on Primary Outcome Measures

#### 3.4.1. Gait Speed

**Figure 3:**
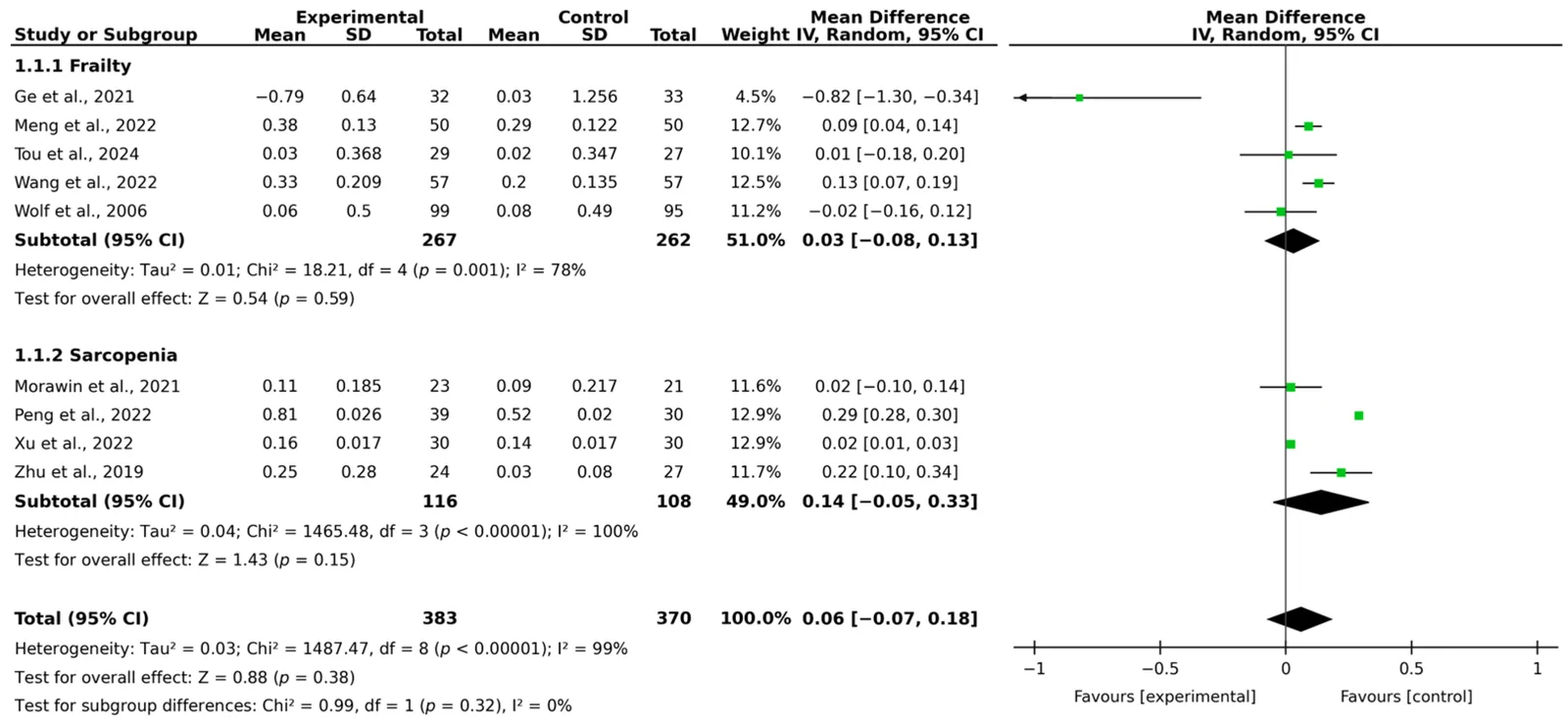
Forest plot of pooled mean differences for frailty and sarcopenia. Effect estimates and 95% confidence intervals were calculated using an inverse-variance random-effects model. Squares represent individual-study estimates, with sizes proportional to study weights; horizontal lines indicate 95% confidence intervals; diamonds represent pooled estimates; and the vertical line indicates no effect. The included studies are reported in References [12,15,19,20,25,26,27,29,33]. CI, confidence interval; IV, inverse variance.

Nine studies reported data on gait speed [12,15,19,20,25,26,27,29,33]. Heterogeneity testing indicated considerable heterogeneity among the nine studies (*p* < 0.01; I^2^ = 99%). Therefore, a random-effects model was used to calculate the pooled effect size. The meta-analysis showed that the post-intervention gait speed in the experimental group was not significantly different from that in the control group (MD = 0.06, 95% CI −0.07 to 0.18, *p* = 0.38) (Figure 3). The subgroup analysis showed that, in both conditions, the improvement in gait speed in the experimental group was not significantly different from that in the control group (*p* > 0.05).

#### 3.4.2. 6MWT

**Figure A1:**
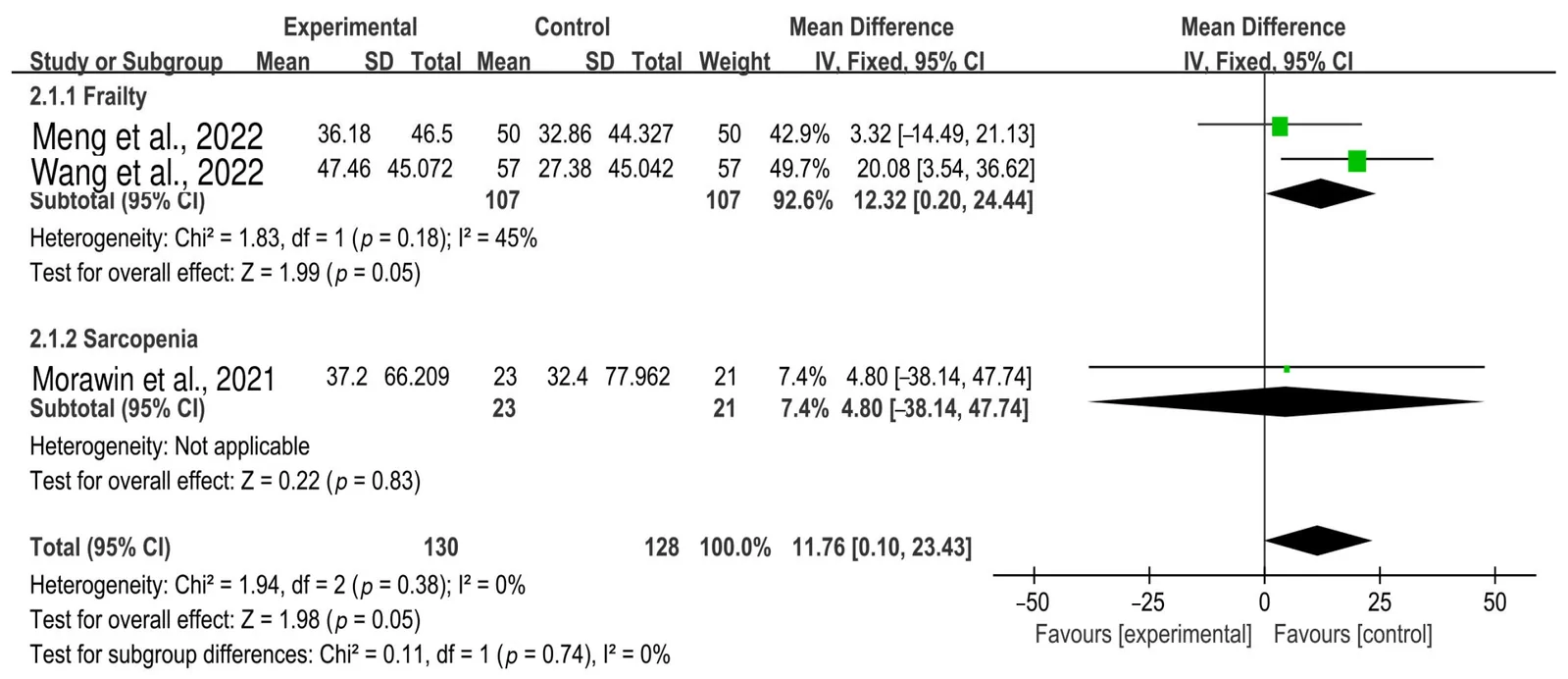
Forest plot comparing post-intervention 6-min walk test (6MWT) performance between the experimental and control groups. Green squares represent individual-study effect estimates, with square size proportional to study weight; horizontal black lines indicate 95% confidence intervals. Black diamonds represent pooled estimates and their 95% confidence intervals. The vertical line at zero indicates no between-group difference. The included studies are reported in References [20,25,27].

Three studies reported data on the 6MWT [20,25,27]. Heterogeneity testing indicated low heterogeneity among the three studies (*p* = 0.38; I^2^ = 0%). Therefore, a fixed-effect model was used for the meta-analysis. The results showed a significant improvement in 6MWT performance in the experimental group compared with the control group after the intervention (MD = 11.76, 95% CI 0.10 to 23.43, *p* = 0.05), suggesting a marginal improvement in aerobic endurance (Figure A1). The sarcopenia subgroup included only one study involving 44 participants. Its effect estimate was highly imprecise, and the 95% CI crossed the line of no effect (MD = 4.80 m, 95% CI −38.14 to 47.74). Therefore, no reliable conclusion regarding the effect of the intervention on 6MWT performance in participants with sarcopenia could be drawn. The test for subgroup differences was not significant (*p* = 0.74).

#### 3.4.3. 30CST

**Figure A2:**
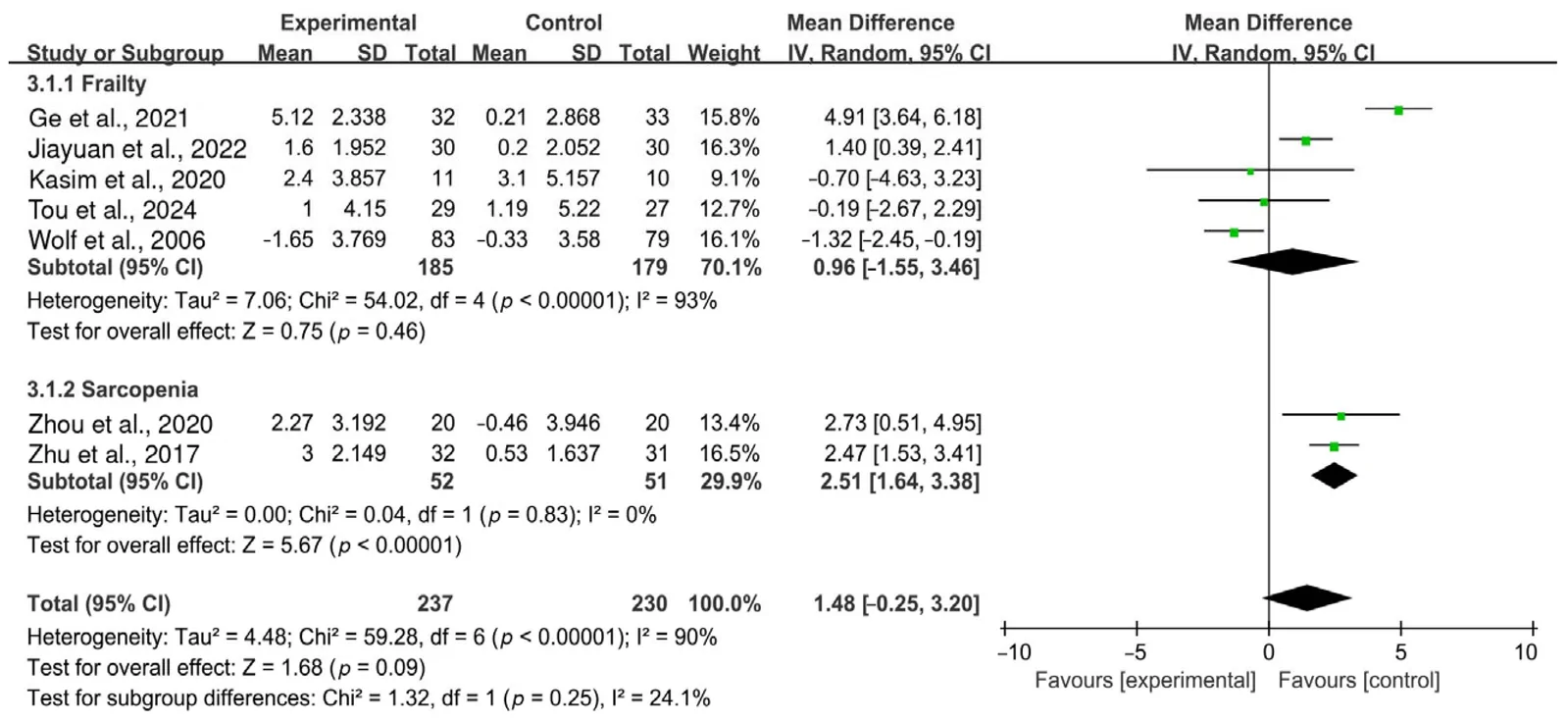
Forest plot comparing post-intervention 30-s chair stand test performance between the experimental and control groups. Green squares represent individual-study effect estimates, with square size proportional to study weight; horizontal black lines indicate 95% confidence intervals. Black diamonds represent pooled estimates and their 95% confidence intervals. The vertical line at zero indicates no between-group difference. The included studies are reported in References [12,14,17,18,19,22,33].

Seven studies reported outcomes on the 30CST Heterogeneity testing indicated substantial heterogeneity across the seven studies (*p* < 0.01; I^2^ = 90%). Therefore, a random-effects model was used for the pooled analysis. Overall, the experimental and control groups did not differ significantly in 30CST performance after the intervention (MD = 1.48, 95% CI −0.25 to 3.20, *p* = 0.09) (Figure A2). In the subgroup analysis, no significant difference was observed among participants with frailty (MD = 0.96, 95% CI −1.55 to 3.46, *p* = 0.46). However, among participants with sarcopenia, the experimental group showed a statistically significant improvement compared with the control group (MD = 2.51, 95% CI 1.64 to 3.38, *p* < 0.01). These findings suggest that the effect of Health Qigong on lower-limb muscle strength may vary according to participant characteristics, with the observed improvement primarily driven by studies involving older adults with sarcopenia.

#### 3.4.4. SST

**Figure A3:**
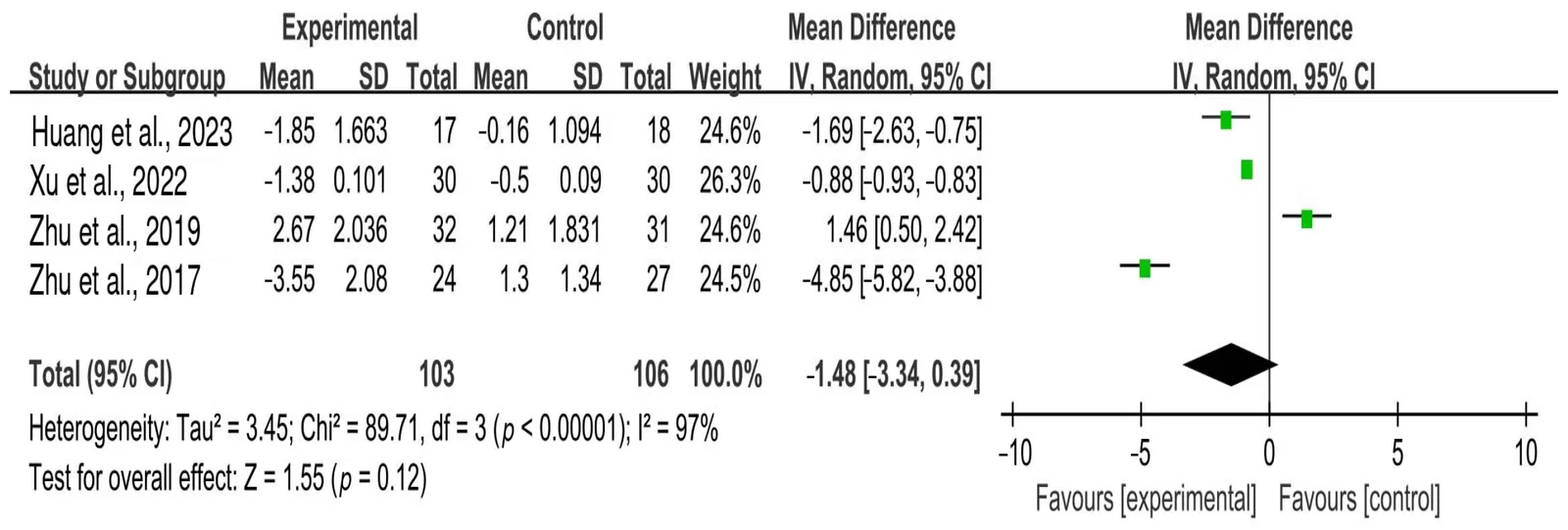
Forest plot comparing post-intervention SST performance between the experimental and control groups. Green squares represent individual-study effect estimates, with square size proportional to study weight; horizontal black lines indicate 95% confidence intervals. The black diamond represents the pooled estimate and its 95% confidence interval. The vertical line at zero indicates no between-group difference. The included studies are reported in References [14,15,29,31].

Four studies reported data on the SST, and all participants in these studies were diagnosed with sarcopenia [14,15,29,31]. Heterogeneity testing indicated considerable heterogeneity among the four studies (*p* < 0.01; I^2^ = 97%). Therefore, a random-effects model was used for the meta-analysis. The results showed that the reduction in SST duration in the experimental group was not significantly different from that in the control group (MD = −1.48, 95% CI −3.34 to 0.39, *p* = 0.12) (Figure A3). This finding suggests that the effect of Health Qigong on SST duration remains inconclusive in older adults with sarcopenia.

#### 3.4.5. TUGT

**Figure 4:**
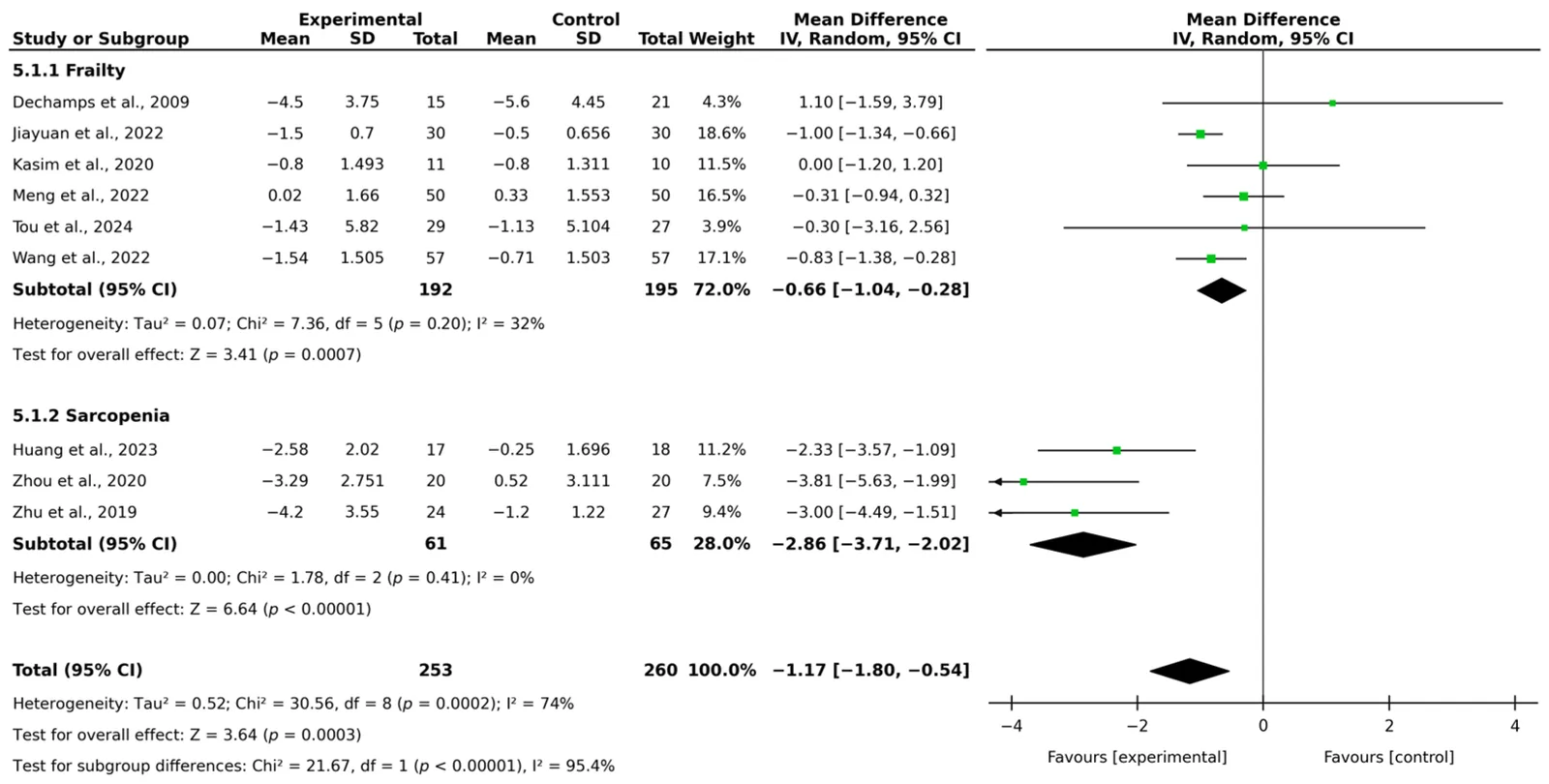
Forest plot of pooled mean differences in frailty and sarcopenia between the experimental and control groups. Green squares represent individual-study effect estimates, with square sizes proportional to study weights. Horizontal black lines indicate the corresponding 95% confidence intervals. Black diamonds represent pooled effect estimates and their 95% confidence intervals for each subgroup and the overall analysis. The vertical line at a mean difference of zero indicates no effect. Negative values favour the experimental group, whereas positive values favour the control group. The included studies are reported in References [13,15,17,18,22,25,27,31,33]. CI, confidence interval; IV, inverse variance.

Nine studies reported data on the TUGT [13,15,17,18,22,25,27,31,33]. Heterogeneity testing indicated substantial heterogeneity among the nine studies (*p* < 0.01; I^2^ = 74%). Therefore, a random-effects model was used for the pooled analysis. The pooled analysis showed that Health Qigong was associated with a shorter TUGT completion time (MD = −1.17 s, 95% CI −1.80 to −0.54). However, substantial heterogeneity was observed (I^2^ = 74%). Within-subgroup heterogeneity was lower in studies involving participants with frailty (I^2^ = 32%) and absent in studies involving participants with sarcopenia (I^2^ = 0%). The larger pooled reduction among participants with sarcopenia (−2.86 s) than among those with frailty (−0.66 s), together with the significant subgroup difference (*p* < 0.001), suggests that the clinical condition may explain part of the observed variability. Differences in baseline functional status, intervention type and dose, comparator conditions, outcome assessment procedures, and study precision may also have contributed. Therefore, the overall pooled effect should be interpreted cautiously and should not be regarded as a uniform treatment effect across all populations and interventions (Figure 4).

#### 3.4.6. SPPB

**Figure 5:**
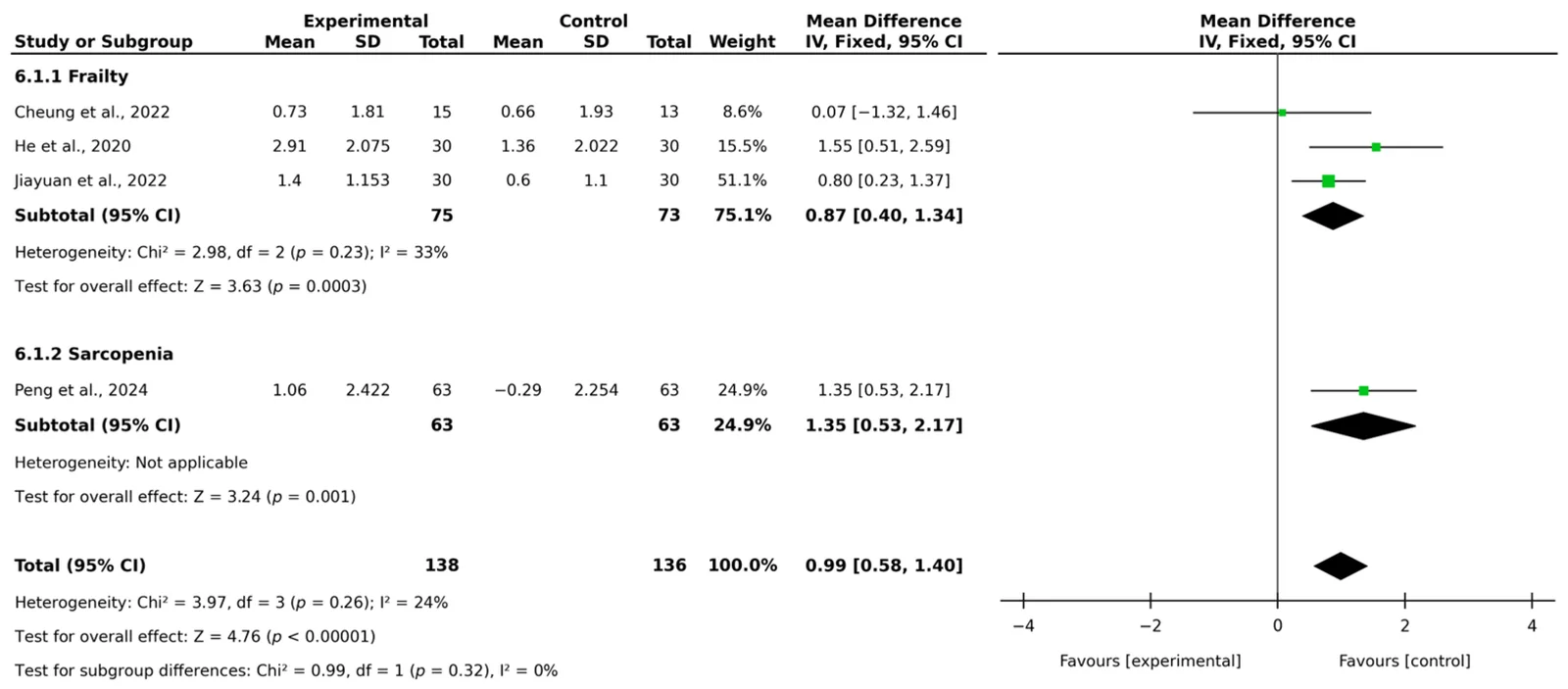
Forest plot of pooled mean differences in frailty and sarcopenia between the experimental and control groups. Effect estimates were pooled using an inverse-variance fixed-effect model. Green squares represent individual-study effect estimates, with square sizes proportional to study weights. Horizontal black lines indicate the corresponding 95% confidence intervals. Black diamonds represent pooled effect estimates and their 95% confidence intervals for each subgroup and the overall analysis. The vertical line at a mean difference of zero indicates no effect. Negative values favour the experimental group, whereas positive values favour the control group. The included studies are reported in References [16,21,22,32]. CI, confidence interval; IV, inverse variance.

Four studies reported data on the SPPB [16,21,22,32]. Heterogeneity testing indicated low heterogeneity among the four studies (*p* = 0.24; I^2^ = 24%). Therefore, a fixed-effect model was used for the meta-analysis. The pooled results showed that the post-intervention SPPB score in the experimental group was significantly higher than that in the control group (MD = 0.99, 95% CI 0.58 to 1.40, *p* < 0.01) (Figure 5), indicating a significant improvement in lower-extremity physical function. The subgroup analysis showed that, in both conditions, the experimental group exhibited significantly greater improvements in SPPB scores than the control group (*p* < 0.05). These findings suggest that Health Qigong effectively improves physical performance in older adults with frailty and those with sarcopenia.

#### 3.4.7. BBS

**Figure A4:**
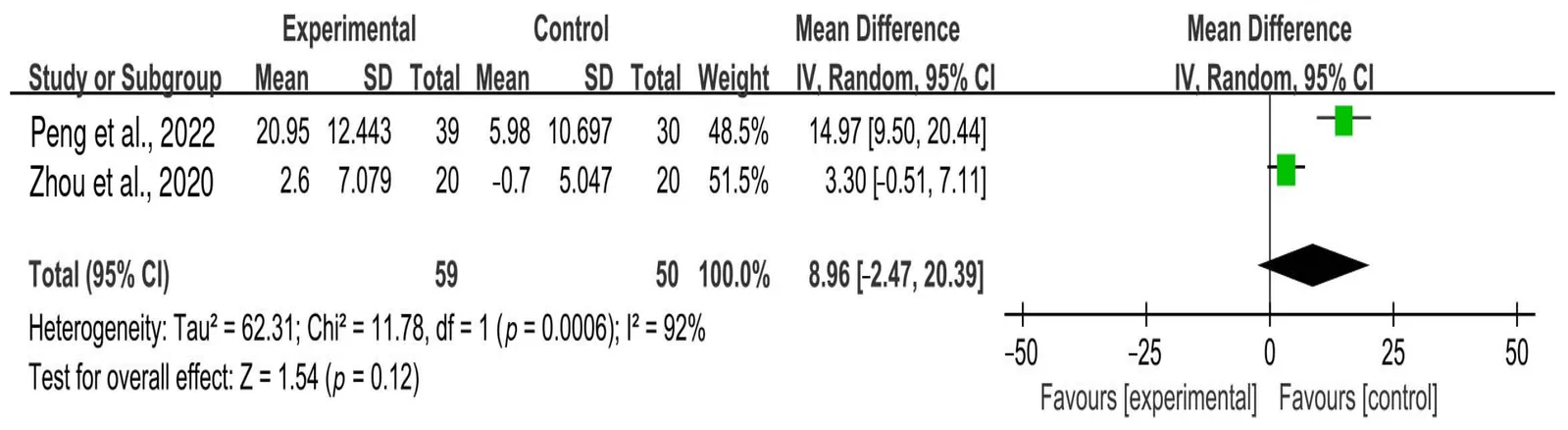
Forest plot comparing post-intervention Berg Balance Scale (BBS) scores between the experimental and control groups. Green squares represent individual-study effect estimates, with square size proportional to study weight; horizontal black lines indicate 95% confidence intervals. The black diamond represents the pooled estimate and its 95% confidence interval. The vertical line at zero indicates no between-group difference. The included studies are reported in References [18,26].

Two studies reported data on the BBS, and all participants in these studies were diagnosed with sarcopenia [18,26]. Heterogeneity testing indicated considerable heterogeneity between the two studies (*p* < 0.01; I^2^ = 92%). Therefore, a random-effects model was used for the pooled analysis. The meta-analysis showed that the post-intervention BBS score in the experimental group was not significantly different from that in the control group (MD = 8.96, 95% CI −2.47 to 20.39, *p* = 0.12) (Figure A4). This finding indicates that the effect of Health Qigong on balance performance remains inconclusive in older adults with sarcopenia.

### 3.5. Meta-Analysis of the Effects of Health Qigong on Secondary Outcome Measures

#### 3.5.1. Muscle Mass

**Figure A5:**
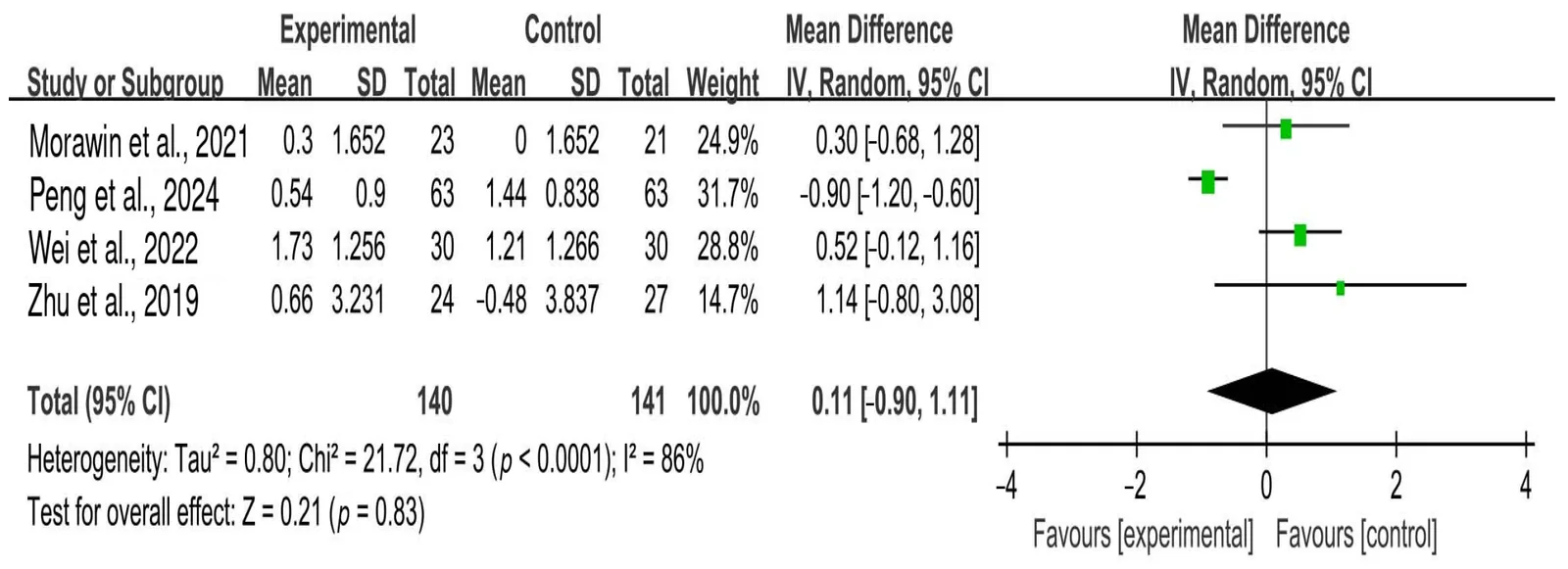
Forest plot comparing post-intervention muscle mass between the experimental and control groups using mean differences. Green squares represent individual-study effect estimates, with square size proportional to study weight; horizontal black lines indicate 95% confidence intervals. The black diamond represents the pooled estimate and its 95% confidence interval. The vertical line at zero indicates no between-group difference. The included studies are reported in References [15,20,28,32].

Four studies reported data on muscle mass, and all participants in these studies were diagnosed with sarcopenia [15,20,28,32]. Heterogeneity testing indicated considerable heterogeneity among the four studies (*p* < 0.01; I^2^ = 86%). Therefore, a random-effects model was used for the meta-analysis. The post-intervention muscle mass in the experimental group was not significantly different from that in the control group (MD = 0.11, 95% CI −0.90 to 1.11, *p* = 0.83) (Figure A5). This finding suggests that Health Qigong did not produce a measurable improvement in muscle mass in older adults with sarcopenia.

#### 3.5.2. Grip Strength

**Figure 6:**
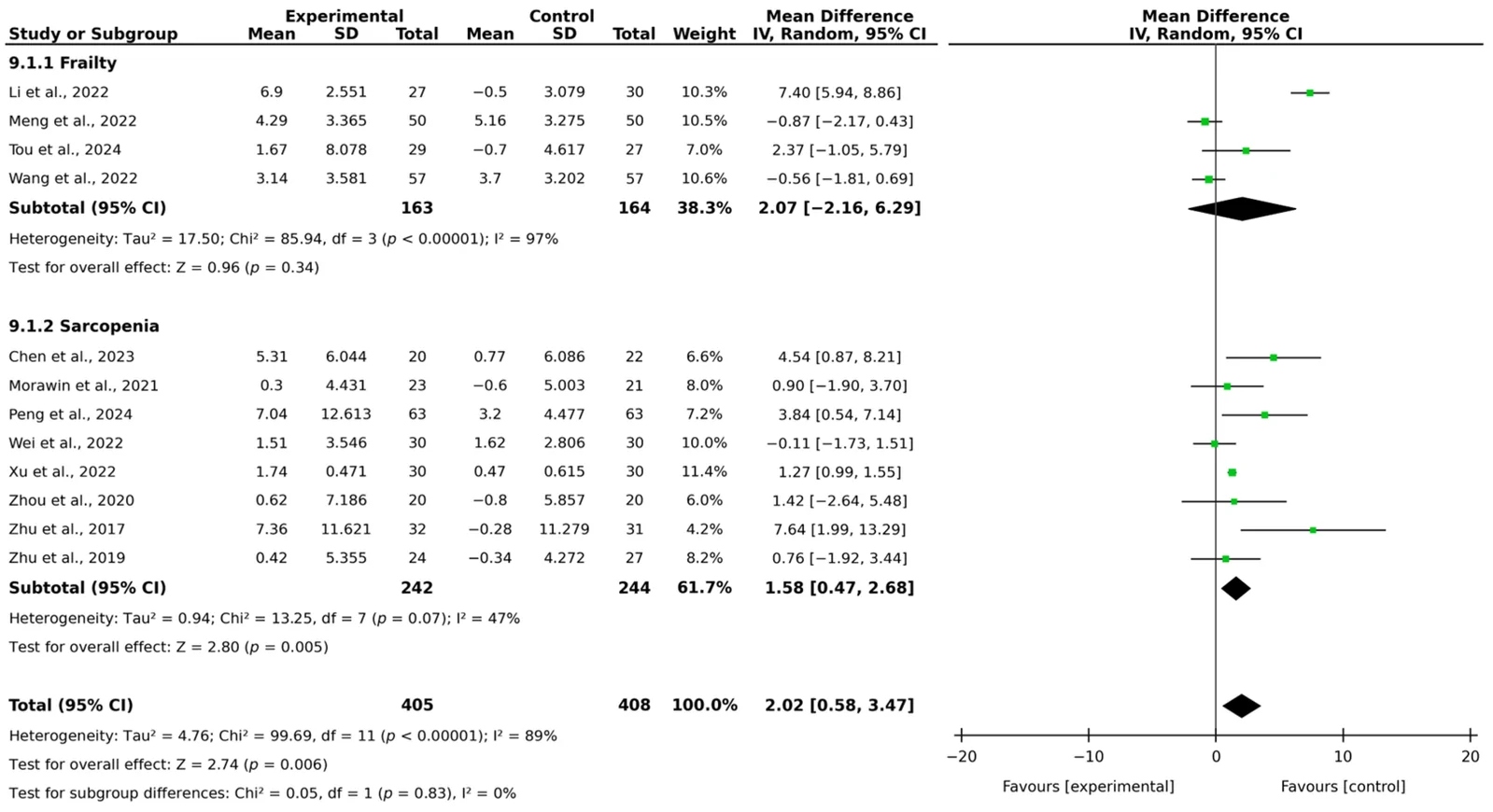
Forest plot of pooled mean differences in frailty and sarcopenia between the experimental and control groups. Effect estimates were pooled using an inverse-variance random-effects model. Green squares represent individual-study effect estimates, with square sizes proportional to study weights. Horizontal black lines indicate the corresponding 95% confidence intervals. Black diamonds represent pooled effect estimates and their 95% confidence intervals for each subgroup and the overall analysis. The vertical line at a mean difference of zero indicates no effect. Negative values favour the experimental group, whereas positive values favour the control group. The included studies are reported in References [14,15,18,20,23,25,27,28,29,30,32,33]. CI, confidence interval; IV, inverse variance.

Twelve studies reported data on grip strength [14,15,18,20,23,25,27,28,29,30,32,33]. Heterogeneity testing indicated considerable heterogeneity among the 12 studies (*p* < 0.01; I^2^ = 89%). Therefore, a random-effects model was used to calculate the pooled effect size. The meta-analysis showed that the post-intervention grip strength in the experimental group was significantly greater than that in the control group (MD = 2.02, 95% CI 0.58 to 3.47, *p* = 0.006) (Figure 6), indicating a significant improvement in upper-limb muscle strength. The subgroup analysis showed that, among participants with frailty, the experimental and control groups did not differ significantly (MD = 2.07, 95% CI −2.16 to 6.29, *p* = 0.34). However, among participants with sarcopenia, the experimental group showed a significant improvement compared with the control group (MD = 1.58, 95% CI 0.47 to 2.68, *p* < 0.01). Overall, these findings suggest that the improvement in grip strength was primarily driven by studies involving participants with sarcopenia. However, the subgroup results should be interpreted cautiously because of the considerable between-study heterogeneity.

#### 3.5.3. FOF

**Figure A6:**
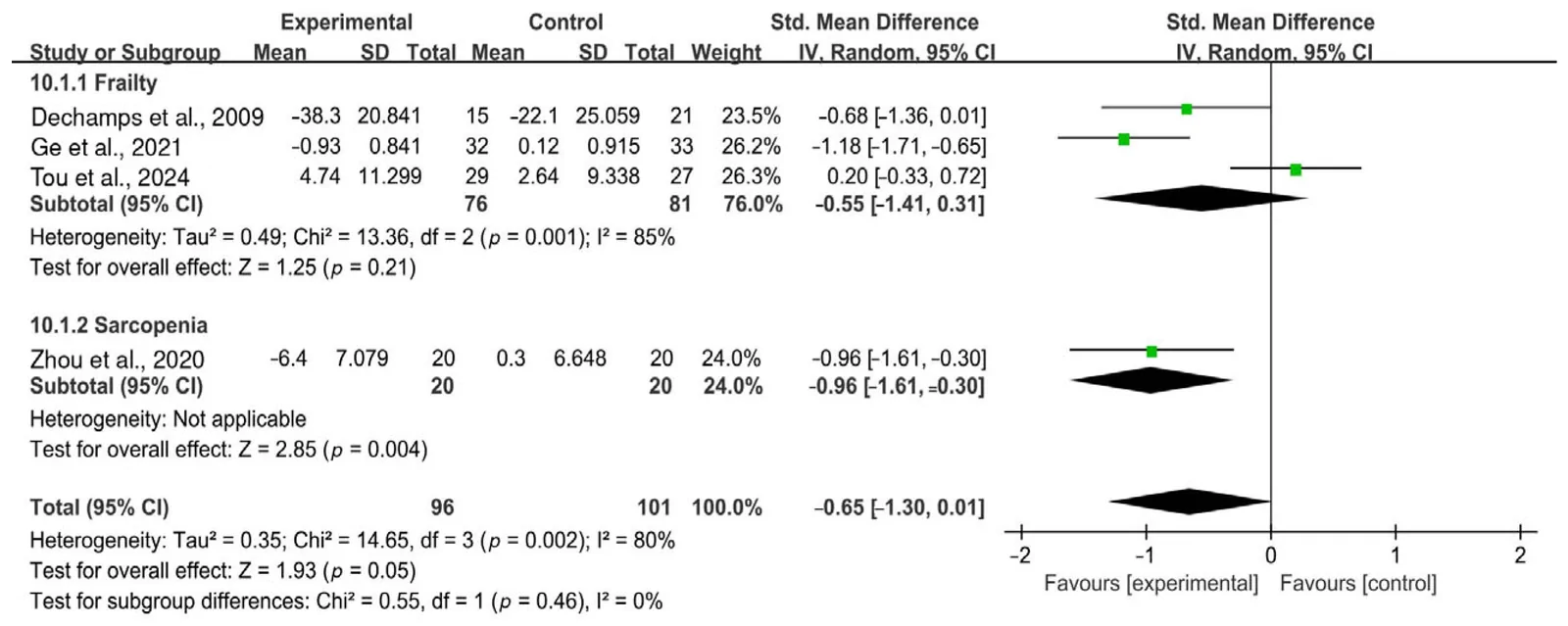
Forest plot comparing post-intervention muscle mass between the experimental and control groups using standardized mean differences. Green squares represent individual-study effect estimates, with square size proportional to study weight; horizontal black lines indicate 95% confidence intervals. Black diamonds represent pooled estimates and their 95% confidence intervals. The vertical line at zero indicates no between-group difference. The included studies are reported in References [13,18,19,33].

Four studies reported data on the FOF [13,18,19,33]. Heterogeneity testing indicated considerable heterogeneity among the four studies (*p* < 0.01; I^2^ = 80%). Therefore, a random-effects model was used. Overall, the experimental group showed a borderline reduction in FOF compared with the control group (SMD = −0.65, 95% CI −1.30 to 0.01, *p* = 0.05), suggesting a potential but uncertain effect (Figure A6). The subgroup analysis showed that, among participants with frailty, no statistically significant difference was observed between the experimental and control groups (SMD = −0.55, 95% CI −1.41 to 0.31, *p* = 0.21). However, among participants with sarcopenia, the experimental group showed a significant reduction in FOF compared with the control group (SMD = −0.96, 95% CI −1.61 to −0.30, *p* < 0.01). These findings suggest that the effect of Health Qigong on FOF may vary according to participant characteristics, with the observed reduction primarily driven by studies involving older adults with sarcopenia.

#### 3.5.4. MMSE

**Figure A7:**
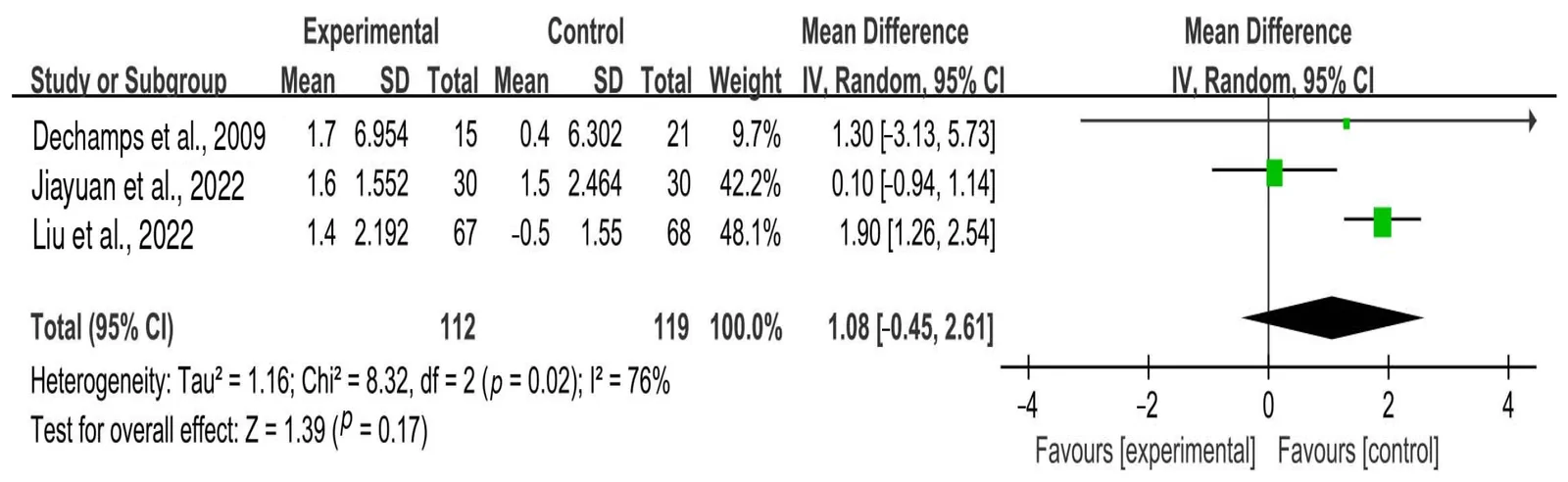
Forest plot comparing post-intervention Mini-Mental State Examination (MMSE) scores between the experimental and control groups. Green squares represent individual-study effect estimates, with square size proportional to study weight; horizontal black lines indicate 95% confidence intervals. The black diamond represents the pooled estimate and its 95% confidence interval. The vertical line at zero indicates no between-group difference. The included studies are reported in References [13,22,24].

Three studies reported data on the MMSE, and all participants in these studies were diagnosed with frailty [13,22,24]. Heterogeneity testing indicated considerable heterogeneity among the three studies (*p* = 0.02; I^2^ = 76%). Therefore, a random-effects model was used for the meta-analysis. The pooled results showed that the post-intervention MMSE score in the experimental group was not significantly different from that in the control group (MD = 1.08, 95% CI −0.45 to 2.61, *p* = 0.17) (Figure A7). This finding suggests that Health Qigong has limited or inconsistent effects on cognitive function in older adults with frailty.

#### 3.5.5. Depressive Symptoms

**Figure A8:**
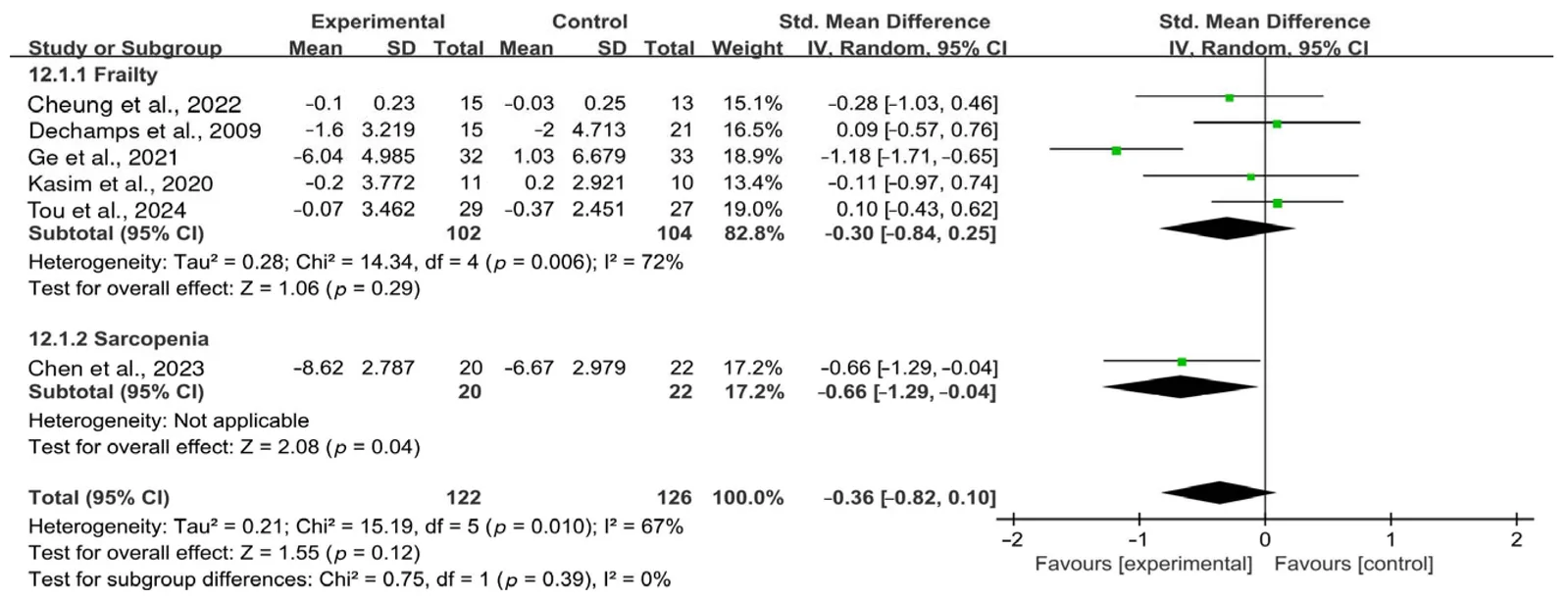
Forest plot comparing post-intervention depressive symptom scores between the experimental and control groups. Green squares represent individual-study effect estimates, with square size proportional to study weight; horizontal black lines indicate 95% confidence intervals. Black diamonds represent pooled estimates and their 95% confidence intervals. The vertical line at zero indicates no between-group difference. The included studies are reported in References [13,17,19,21,30,33].

Six studies reported data on depressive symptoms [13,17,19,21,30,33]. Heterogeneity testing indicated substantial heterogeneity among the six studies (*p* = 0.01; I^2^ = 67%). Therefore, a random-effects model was used. Overall, the experimental and control groups did not differ significantly in depressive symptoms (SMD = −0.36, 95% CI −0.82 to 0.10, *p* = 0.12) (Figure A8). The subgroup analysis showed that Health Qigong did not have a statistically significant effect in participants with frailty (SMD = −0.30, 95% CI −0.84 to 0.25, *p* = 0.29). However, among participants with sarcopenia, Health Qigong produced a statistically significant improvement in depressive symptoms (SMD = −0.66, 95% CI −1.29 to −0.04, *p* = 0.04). These findings suggest that the effect of Health Qigong on depressive symptoms may vary across participant populations, with the observed improvement potentially driven by studies involving participants with sarcopenia. However, the subgroup findings should be interpreted with caution given the non-significant overall effect.

#### 3.5.6. QOL

**Figure A9:**
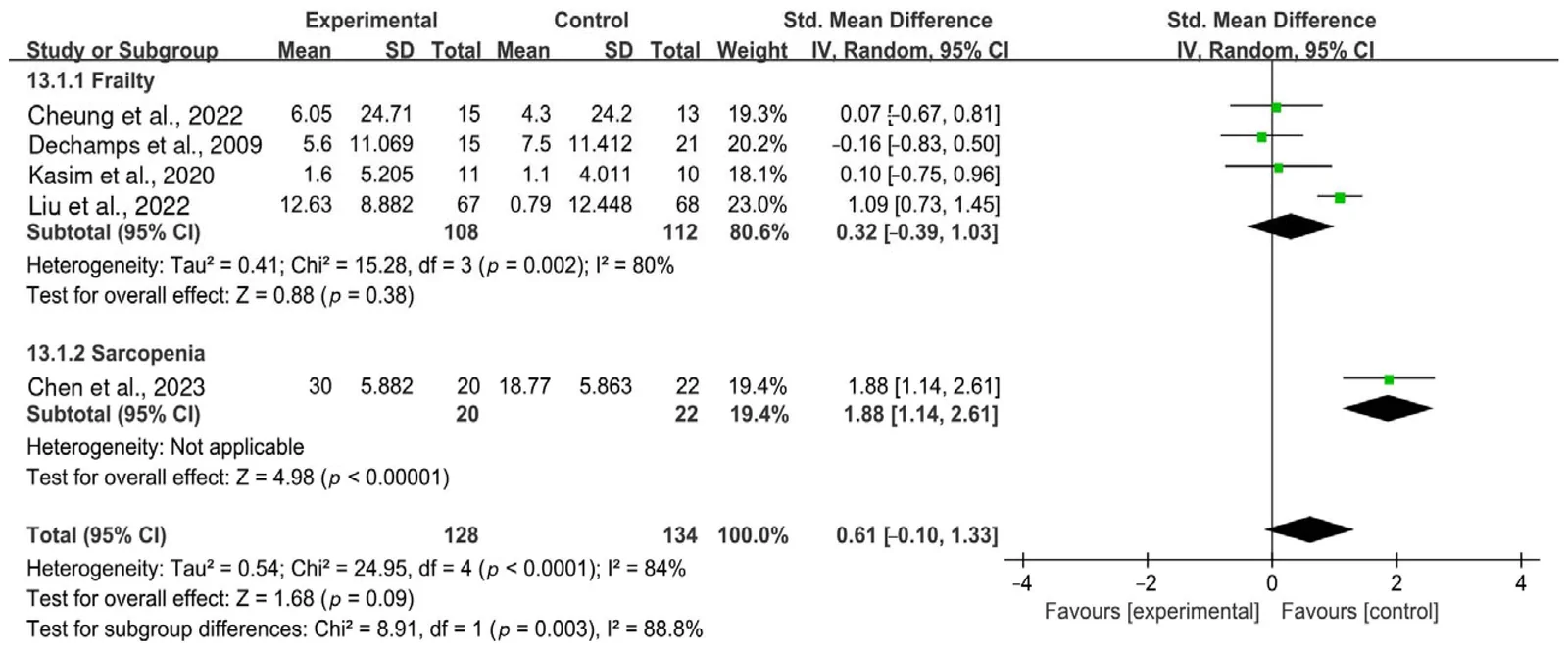
Forest plot comparing post-intervention quality-of-life (QOL) scores between the experimental and control groups. Green squares represent individual-study effect estimates, with square size proportional to study weight; horizontal black lines indicate 95% confidence intervals. Black diamonds represent pooled estimates and their 95% confidence intervals. The vertical line at zero indicates no between-group difference. The included studies are reported in References [13,17,21,24,30].

Five studies reported data on QOL [13,17,21,24,30]. Heterogeneity testing indicated considerable heterogeneity among the five studies (*p* < 0.01; I^2^ = 84%). Therefore, a random-effects model was used for the meta-analysis. The post-intervention QOL score in the experimental group was not significantly different from that in the control group (SMD = 0.61, 95% CI −0.10 to 1.33, *p* = 0.09) (Figure A9). The subgroup analysis showed that, among participants with frailty, the experimental group showed no significant improvement in QOL compared with the control group (SMD = 0.32, 95% CI −0.39 to 1.03, *p* = 0.38). In contrast, among participants with sarcopenia, the experimental group showed a statistically significant improvement in QOL (SMD = 1.88, 95% CI 1.14 to 2.61, *p* < 0.01). These findings suggest that Health Qigong may improve quality of life in older adults with sarcopenia.

#### 3.5.7. MBI

**Figure A10:**
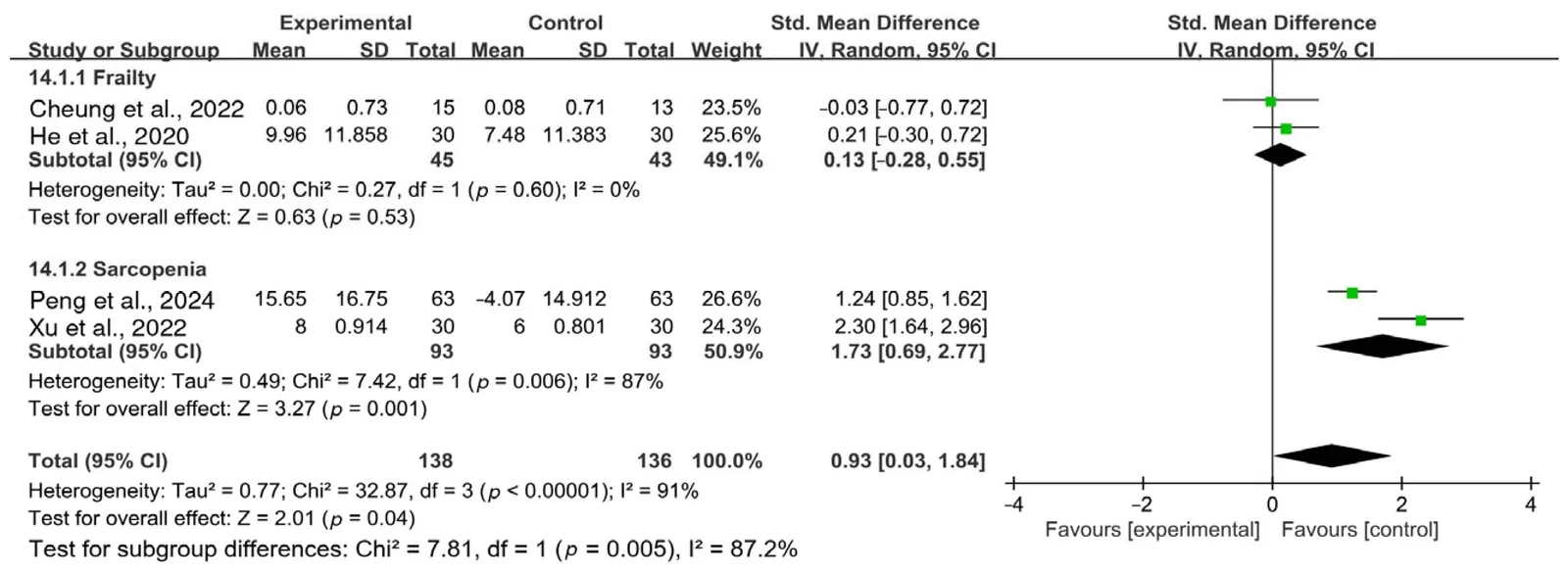
Forest plot comparing post-intervention Modified Barthel Index (MBI) scores between the experimental and control groups. Green squares represent individual-study effect estimates, with square size proportional to study weight; horizontal black lines indicate 95% confidence intervals. Black diamonds represent pooled estimates and their 95% confidence intervals. The vertical line at zero indicates no between-group difference. The included studies are reported in References [16,21,29,32].

Four studies reported data on MBI scores [16,21,29,32]. Heterogeneity testing indicated considerable heterogeneity among the four studies (*p* < 0.01; I^2^ = 91%). Therefore, a random-effects model was used. Overall, the experimental group showed significantly greater post-intervention MBI scores than the control group (SMD = 0.93, 95% CI 0.03 to 1.84, *p* = 0.04) (Figure A10), suggesting a beneficial effect on activities of daily living. The subgroup analysis showed that, among participants with frailty, no statistically significant difference was observed between the experimental and control groups (SMD = 0.13, 95% CI −0.28 to 0.55, *p* = 0.53). However, among participants with sarcopenia, the experimental group showed a significant improvement (SMD = 1.73, 95% CI 0.69 to 2.77, *p* < 0.01). These findings suggest that Health Qigong may improve functional independence, with the effect appearing to be more pronounced in older adults with sarcopenia. However, the subgroup findings should be interpreted with caution because of the considerable between-study heterogeneity and the limited number of included studies.

### 3.6. Publication Bias

**Figure A11:**
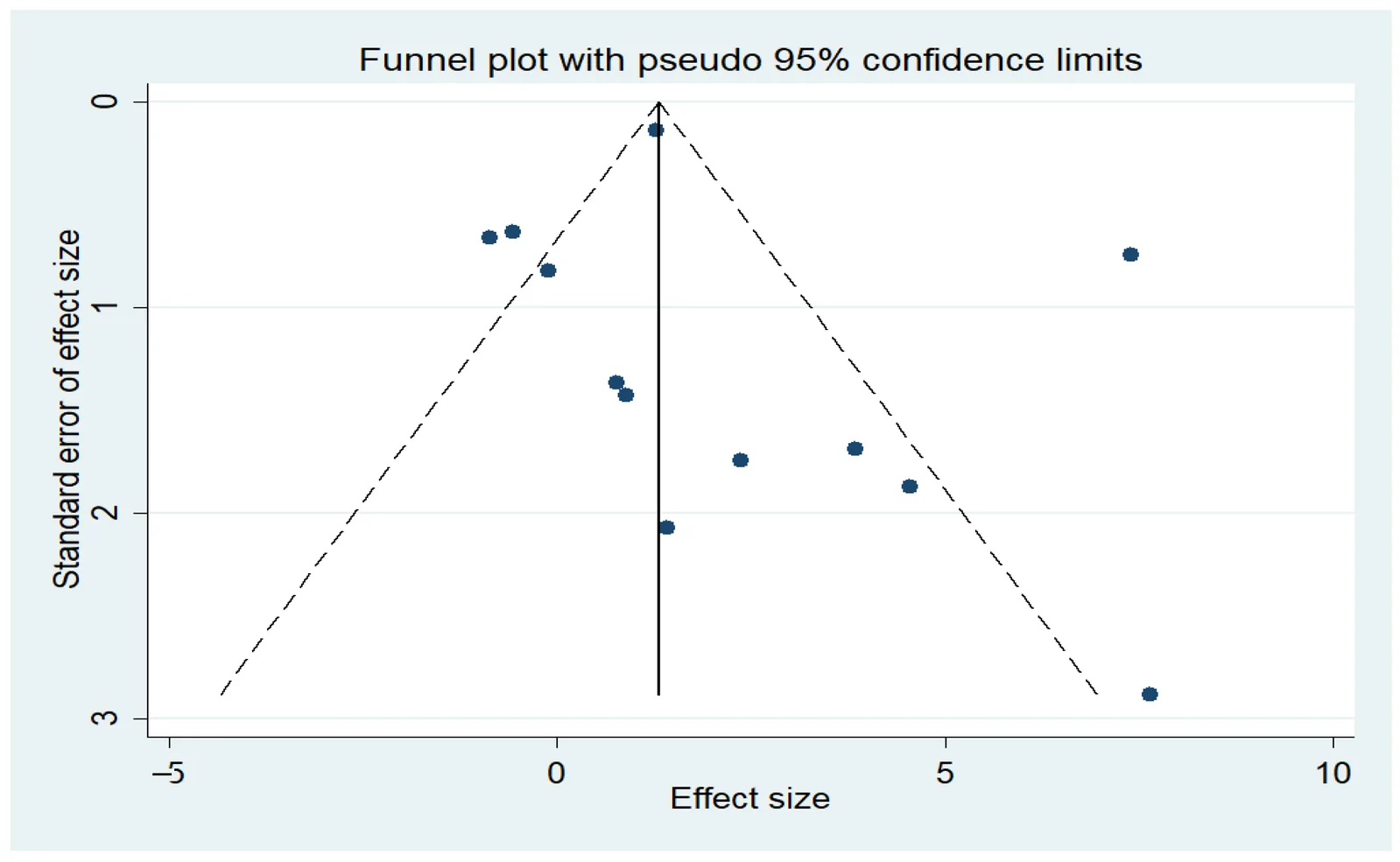
Funnel plot assessing publication bias for grip strength. Blue points represent individual studies; the solid vertical line indicates the pooled effect estimate, and the dashed diagonal lines indicate pseudo 95% confidence limits. Visual asymmetry was examined as an indicator of possible small-study effects or publication bias. The included studies are reported in References [14,15,18,20,23,25,27,28,29,30,32,33].

Grip strength was the only outcome represented by at least 10 studies (k = 12) and was therefore the only outcome for which funnel-plot and Egger-test assessments were conducted. The funnel plot appeared slightly asymmetric (Figure A11), but Egger’s regression test did not show statistical evidence of small-study effects (*p* = 0.54). This result should not be interpreted as proof that publication bias was absent, because the test had limited power with only 12 studies and the analysis showed substantial heterogeneity (I^2^ = 89%).

### 3.7. Meta-Regression

Exploratory univariable meta-regression (Table A2) was restricted to grip strength because it was the only outcome reported in at least 10 studies. Neither diagnostic group (sarcopenia versus frailty: β = 0.09 kg, 95% CI −3.86 to 4.04; *p* = 0.960) nor intervention duration (β per week = −0.023 kg, 95% CI −0.211 to 0.166; *p* = 0.793) was associated with the observed treatment effect. Neither moderator explained the between-study variance (R^2^ = 0%), and substantial residual heterogeneity remained in both models (residual I^2^ ≈ 89%). Therefore, the available study-level variables did not explain the heterogeneity observed in the grip-strength analysis.

## 4. Discussion

Aging is a multisystem physiological and pathological process characterized by two major degenerative syndromes: sarcopenia and frailty [34]. From a clinical perspective, sarcopenia is primarily characterized by a triad of skeletal muscle mass loss, reduced muscle strength, and impaired physical function. In contrast, frailty is associated with more extensive functional impairments. It involves multisystem decline manifested by, but not limited to, reduced muscle strength, impaired balance, and decreased exercise tolerance [35]. Both conditions are associated with imbalances between muscle protein synthesis and degradation, hormonal alterations, chronic inflammation, and degeneration of the neuromuscular junctions [36,37]. Clinically, they often lead to adverse outcomes such as falls, disability, fractures, and mortality [38]. These degenerative syndromes are strongly associated with poor clinical outcomes and significantly increase the risks of falls and all-cause mortality in older adults.

As an intervention that combines breathing regulation, mental focus, and physical movement [39], Health Qigong has demonstrated unique advantages in the rehabilitation of older adults with sarcopenia and frailty, distinguishing it from conventional aerobic and resistance training. Biomechanical analyses have shown that representative Health Qigong forms, including Baduanjin, Wuqinxi, Yijinjing, and Tai Chi, share common movement patterns characterized by multiplanar compound movements involving coordinated activity across the sagittal, coronal, and transverse planes. These movement patterns effectively activate the deep stabilizing muscles and improve joint range of motion. During postural transitions, concentric–eccentric contraction sequences enhance muscle fiber recruitment efficiency, thereby promoting improvements in muscular endurance and explosive strength, particularly in the lower limbs and core [40,41]. In addition, synchronizing slow breathing (approximately six breaths per minute) with gentle movement patterns rooted in traditional Chinese medicine increases the root mean square of successive differences in heart rate variability, reflecting enhanced parasympathetic activity. This modulation of autonomic balance is consistent with the traditional Chinese medicine principle of the “integration of form and Qi” [42]. From an implementation perspective, Health Qigong requires no specialized equipment or facilities, and its low-impact, low-risk nature makes it particularly suitable for community-dwelling older adults. Moreover, it has been associated with higher adherence than conventional resistance training, particularly in home- and community-based settings [33,43].

Overall, Health Qigong has significant clinical value in the management of sarcopenia and frailty. It exerts beneficial effects through multiple mechanisms, including musculoskeletal optimization, autonomic regulation, and modulation of metabolic pathways.

### 4.1. Integrated Interpretation of the Effects of Health Qigong

Health Qigong significantly improved functional muscle strength in participants with sarcopenia and frailty, as evidenced by a significantly shorter post-intervention TUGT duration in the experimental group than in the control group (MD = −1.17, 95% CI −1.80 to −0.54, *p* < 0.01). This effect may be attributed to the movement-specific activation of key muscle groups. For example, the “Deer Resisting” posture in Wuqinxi, which involves lateral lunges, targets the hip abductor muscles [44]. The “Brush Knee and Twist Step” movement in Tai Chi enhances dorsiflexion–plantarflexion coordination through dynamic weight shifting. The “Golden Rooster Stands on One Leg” posture improves static single-leg support and strengthens lower-limb muscular endurance [45]. Furthermore, the “Pulling Nine Oxen by the Tail” movement in Yijinjing reinforces core muscle co-contraction through anti-rotational trunk training [46]. However, no significant differences were observed in post-intervention gait speed between the experimental and control groups in participants with sarcopenia and frailty (MD = 0.06, 95% CI −0.07 to 0.18, *p* = 0.38). Additionally, post-intervention SST performance did not differ significantly between the experimental and control groups in participants with sarcopenia (MD = −1.48, 95% CI −3.34 to 0.39, *p* = 0.12). These findings suggest that improvements in mobility and explosive strength may be selective and dependent on specific motor patterns. The SST findings showed considerable heterogeneity across the four trials (I^2^ = 97%). This heterogeneity may reflect differences in participant age and baseline functional status, intervention modality and duration, control conditions, and SST assessment protocols. The small sample sizes may also have increased susceptibility to random variation. Consequently, the non-significant pooled estimate should be interpreted as inconclusive rather than as evidence of a consistently ineffective intervention.

Among the secondary outcomes, although skeletal muscle mass did not improve significantly (MD = 0.11, 95% CI −0.90 to 1.11, *p* = 0.83), participants with sarcopenia in the experimental group showed significantly greater post-intervention grip strength than those in the control group (MD = 1.58, 95% CI 0.47 to 2.68, *p* < 0.01). This finding suggests that improvements in neuromuscular control may precede morphological changes such as muscle fiber hypertrophy, indicating a temporal dissociation between functional adaptation and structural remodeling. Therefore, longer intervention durations may produce measurable improvements in muscle mass.

Health Qigong was associated with significantly greater post-intervention 6MWT performance in participants with frailty (MD = 12.32, 95% CI 0.20 to 24.44, *p* = 0.05), whereas it was associated with significantly greater post-intervention 30CST performance in participants with sarcopenia (MD = 2.51, 95% CI 1.64 to 3.38, *p* < 0.01). These findings suggest that Health Qigong may exert differentiated physiological effects through movement–metabolism coupling.

Biologically, these findings may be explained by the postural elements embedded within specific Health Qigong routines. For example, the “Bear Swaying” movement in Wuqinxi, which involves lateral trunk flexion, activates the core musculature. When combined with the semicircular stepping patterns used in Tai Chi, it may enhance quadriceps activation during concentric contractions, thereby optimizing glycogenolysis as an energy-producing process [47]. Additionally, the “Lifting One Arm to Regulate the Spleen and Stomach” movement in Baduanjin may stimulate Qi and blood circulation along the liver and spleen meridians, potentially increasing mitochondrial respiratory chain complex activity and improving energy metabolism in older adults with frailty [48].

Notably, the more pronounced improvement in post-intervention 30CST performance among participants with sarcopenia may be closely related to specific biomechanical features of the exercises. For instance, the “Green Dragon Extends its Claws” movement in Yijinjing promotes isometric contraction of the core muscles during lateral spinal flexion to maintain trunk stability, whereas the “Kick with Heel” movement in Tai Chi enhances lower-limb explosive strength through dynamic single-leg support. In contrast, the borderline improvement in post-intervention 6MWT performance among participants with frailty may reflect a narrower window of metabolic adaptability resulting from multisystem decline. These findings suggest that more targeted Health Qigong modifications may be needed to overcome this physiological compensation threshold.

Health Qigong was associated with significantly higher post-intervention SPPB scores in participants with sarcopenia and frailty than in the control group (MD = 0.99, 95% CI 0.58 to 1.40, *p* < 0.01). This functional improvement may be related to the neuropsychological coupling embedded in Health Qigong movements. For example, the “Tiger Pounce” posture in Wuqinxi, which involves trunk flexion and extension, enhances dynamic spinal stability, while the “Seven Heels Lifting” movement in Baduanjin may stimulate the plantar fascia–cerebellum pathway through heel raises, thereby improving postural control [49]. However, Health Qigong was not associated with significantly higher post-intervention BBS scores in participants with sarcopenia than in the control group (MD = 8.96, 95% CI −2.47 to 20.39, *p* = 0.12). This finding may be attributed to the emphasis of the BBS on static balance assessment, whereas Health Qigong primarily emphasizes dynamic balance and coordinated movement patterns.

Among the secondary outcomes, only one study reported a significant improvement in QOL among participants with sarcopenia following Health Qigong (SMD = 1.88, 95% CI 1.14 to 2.61, *p* < 0.01). Therefore, the certainty of this evidence remains limited and requires further verification. In contrast, other psychosocial outcomes, including FOF (SMD = −0.96, 95% CI −1.61 to −0.30, *p* < 0.01), depressive symptoms (SMD = −0.66, 95% CI −1.29 to −0.04, *p* = 0.04), and MBI scores (SMD = 1.73, 95% CI 0.69 to 2.77, *p* < 0.01), showed significantly better post-intervention outcomes in participants with sarcopenia who received Health Qigong than in the control group. These findings highlight the movement–mind integration effects of Health Qigong. Specifically, the abdominal massage performed during the “Bear Swaying” movement in Wuqinxi, combined with reverse abdominal breathing, may increase vagal tone. The extended-arm posture in the “White Crane Spreads its Wings” movement in Tai Chi may enhance spatial orientation through visual and vestibular feedback, potentially reducing anxiety. By comparison, Health Qigong was not associated with significantly higher post-intervention MMSE scores in participants with frailty than in the control group (MD = 1.08, 95% CI −0.45 to 2.61, *p* = 0.17). This finding suggests that cognitive decline in this population may be driven by more complex neuropathological processes, such as hippocampal atrophy and β-amyloid accumulation. Therefore, motor training alone may need to be supplemented with cognitive tasks to achieve measurable cognitive benefits [50].

Although this meta-analysis did not directly evaluate inflammatory biomarkers, previous studies have suggested that Health Qigong contributes to reducing chronic systemic inflammation. “Inflammaging”—a state of persistent, low-grade inflammation—is now widely recognized as a key driver of age-related syndromes, including frailty [51]. Individuals with frailty have been found to exhibit higher levels of inflammatory markers, particularly C-reactive protein and interleukin-6 (IL-6), than those without frailty [52]. Pro-inflammatory cytokines, such as IL-6 and tumor necrosis factor-alpha, impair muscle protein synthesis and promote protein catabolism, thereby contributing to the loss of muscle mass and strength [52].

Mechanistic studies suggest that Health Qigong enhances vagal modulation and activates the “cholinergic anti-inflammatory pathway,” thereby inhibiting the release of pro-inflammatory cytokines. Additionally, skeletal muscle functions as an endocrine organ during exercise by secreting various anti-inflammatory myokines, most notably IL-6. Unlike persistently elevated circulating IL-6 concentrations, which exert pro-inflammatory effects, the transient release of IL-6 from contracting skeletal muscle during exercise can suppress the production of tumor necrosis factor-alpha and promote the release of anti-inflammatory cytokines, such as IL-10, thereby contributing to systemic immune modulation [53,54]. Overall, these findings suggest that the combination of neuromodulatory and exercise-induced anti-inflammatory mechanisms associated with Health Qigong may delay or attenuate the progression of inflammaging.

### 4.2. Major Findings and Improvements in Specific Outcomes

The GRADE assessment indicated low-certainty evidence for the pooled effects on TUGT performance, SPPB scores, and grip strength and very-low-certainty evidence for gait speed and quality of life. Accordingly, the statistically significant estimates for TUGT, SPPB, and grip strength should be interpreted as possible benefits rather than established effects. The favorable estimates for other outcomes, including the 6MWT and MBI, were not included among the five critical GRADE outcomes and should be considered exploratory. Post-intervention TUGT duration was significantly shorter in the Health Qigong group than in the control group (MD = −1.17, 95% CI −1.80 to −0.54, *p* < 0.01), indicating that Health Qigong may improve balance and mobility. The TUGT is widely used to assess both domains. The physical postures and coordinated movements involved in Health Qigong, such as the animal-mimicking exercises in Wuqinxi and the stretching movements in Yijinjing, may improve lower-limb strength and coordination. Notably, subgroup findings showed significantly greater post-intervention TUGT performance in both the sarcopenia and frailty groups. Mechanistically, Health Qigong movements may stimulate the vestibulospinal reflex pathway, improve vestibular function, and promote neuroplasticity, thereby enhancing postural control [55]. Furthermore, physical activity has been shown to modulate irisin secretion, which may improve bone health and mobility in aging models [56].

Grip strength, an indicator of upper-limb muscle strength, showed significantly greater post-intervention values in the Health Qigong group than in the control group (MD = 2.02, 95% CI 0.58 to 3.47, *p* < 0.01). This effect may be attributed to exercise-induced activation of the AKT/mTOR pathway, which promotes muscle protein synthesis and growth, thereby improving muscular strength and endurance [57].

Health Qigong was associated with significantly greater post-intervention 6MWT performance than the control intervention (MD = 11.76, 95% CI 0.10 to 23.43, *p* = 0.05). This finding suggests that Health Qigong may improve aerobic capacity, with particularly pronounced effects in participants with frailty. Compared with conventional aerobic exercise, Health Qigong is characterized by low-impact, whole-body coordinated movements, allowing gradual improvements in aerobic fitness and overall health without imposing excessive physical stress. Through prolonged low-intensity practice, it may improve mitochondrial function and increase mitochondrial content, both of which are essential for enhancing aerobic endurance. Health Qigong practice may increase intracellular adenosine monophosphate (AMP) levels in skeletal muscle cells, thereby activating the AMP-activated protein kinase (AMPK) signaling pathway [58]. AMPK is a key regulator of energy metabolism that promotes fatty acid oxidation and glucose metabolism and enhances mitochondrial biogenesis and function, thereby improving aerobic metabolic capacity [59]. Moreover, during Health Qigong practice, deep and slow abdominal breathing may enhance pulmonary gas exchange efficiency, improve cardiovascular function, and promote nitric oxide synthesis. These effects facilitate vasodilation, improve blood circulation, and enhance oxygen delivery and utilization [60]. Deep breathing and relaxation training may also activate the parasympathetic nervous system, thereby facilitating heart rate regulation and improving systolic and diastolic cardiac function [61]. Finally, long-term Health Qigong practice may activate the Nrf2 signaling pathway, a key transcription factor regulating the antioxidant stress response. Activation of Nrf2 promotes the expression of antioxidant enzymes, such as superoxide dismutase, thereby reducing oxidative cellular damage, protecting muscle tissue from oxidative stress, and ultimately enhancing muscular endurance and function [62].

Health Qigong was associated with significantly higher post-intervention SPPB scores than the control group (MD = 0.99, 95% CI 0.58 to 1.40, *p* < 0.01), reflecting an overall improvement in physical function. Notably, subgroup findings showed significantly higher post-intervention SPPB scores in both the sarcopenia and frailty groups. These improvements may be mediated by gains in muscular strength and balance [63].

Health Qigong was associated with significantly higher post-intervention MBI scores than the control group (SMD = 0.93, 95% CI 0.03 to 1.84, *p* = 0.04), indicating improved performance of activities of daily living. This finding is consistent with the observed improvements in neuromuscular coordination, postural stability, cardiopulmonary endurance, and mental well-being. Collectively, these improvements may contribute to greater functional independence in daily life [64].

### 4.3. Limitations of This Study and Recommendations for Future Studies

This study systematically elucidated the multidimensional benefits of Health Qigong in the rehabilitation of older adults with sarcopenia and frailty. Nonetheless, several limitations should be acknowledged. First, the current body of evidence on Health Qigong remains limited in quantity and methodological consistency. The included studies vary in terms of intervention modalities and durations, and the dose–response relationship of specific movement combinations remains unclear. Moreover, most studies lack high-level imaging or biomolecular evidence to support their findings. Second, the sample sizes of the included clinical trials were generally small (median: 60 participants), with 76.2% of the trials conducted in China. This geographic concentration limits cross-cultural generalizability and statistical power, thereby reducing the robustness and applicability of the conclusions and constraining subgroup analyses. Third, there is a lack of standardization in the implementation of Qigong, particularly in terms of frequency, intensity, and duration. Additionally, notable heterogeneity exists among Qigong styles and execution protocols across studies. This contributes to inconsistency in outcomes and a limited understanding of underlying biological mechanisms. Finally, the review may be subject to publication bias, as studies with positive outcomes are more likely to be published. Several pooled analyses showed substantial or considerable between-study heterogeneity, including gait speed (I^2^ = 99%), the 30 s chair stand test (I^2^ = 90%), sit-to-stand time (I^2^ = 97%), the Berg Balance Scale (I^2^ = 92%), muscle mass (I^2^ = 86%), grip strength (I^2^ = 89%), fear of falling (I^2^ = 80%), quality of life (I^2^ = 84%), and the Modified Barthel Index (I^2^ = 91%). Potential sources include differences in diagnostic criteria and baseline severity, exercise form and dose, co-interventions, comparator conditions, outcome instruments, and assessment timing. Random-effects models and condition-based subgroup analyses were used, and exploratory meta-regression was performed for grip strength; however, substantial residual heterogeneity remained. Consequently, these pooled estimates should be interpreted as average effects across clinically diverse studies rather than as uniform treatment effects. The high heterogeneity reduces confidence in the magnitude and generalizability of both statistically significant and nonsignificant findings.

To address these limitations, future research should incorporate dose–response meta-analyses to quantify the associations between training parameters (e.g., frequency, intensity, and duration) and clinical outcomes. Additionally, multi-omics approaches (e.g., muscle proteomics and gut microbiome metabolomics) should be employed to elucidate the underlying mechanistic pathways. From a clinical perspective, large-scale, multicenter RCTs conducted across diverse cultural settings (≥5 regions) are needed to improve external validity and facilitate the development of precision-guided intervention strategies for determining optimal individualized training doses. Regarding translational application, Health Qigong may represent a potentially useful complementary intervention alongside established care for older adults with sarcopenia or frailty. However, given the heterogeneity, imprecision, small and geographically concentrated samples, variability in intervention protocols, and risk-of-bias concerns in the current evidence base, the available evidence is insufficient to support its routine implementation or inclusion in clinical guidelines. Further large, rigorously conducted multicenter RCTs using standardized intervention protocols and longer follow-up periods are required to confirm its clinical effectiveness, durability, safety, and generalizability before firm recommendations for routine clinical practice can be made. If these benefits are confirmed, the feasibility and accessibility of community-based Health Qigong programs could then be evaluated. These efforts will help clarify the mechanisms and therapeutic value of Health Qigong in the management of sarcopenia and frailty, ultimately providing a stronger scientific foundation for its clinical application.

## 5. Conclusions

Low-certainty evidence suggests that Health Qigong may produce small improvements in post-intervention TUGT performance, SPPB scores, and grip strength in older adults with sarcopenia or frailty. Very-low-certainty evidence leaves its effects on gait speed and quality of life uncertain. Favorable findings for the 6MWT and MBI were not included among the critical GRADE outcomes and should be regarded as exploratory.

Confidence in the current estimates is limited by risk-of-bias concerns, substantial between-study heterogeneity for several outcomes, imprecision, small and geographically concentrated samples, and variation in intervention protocols and comparator conditions. Therefore, the available evidence remains insufficient to support routine implementation or firm condition-specific recommendations. Larger, rigorously conducted multicenter trials using standardized intervention protocols, clinically meaningful outcome thresholds, and longer follow-up periods are needed.

## Figures and Tables

**Table 1:** PICOS eligibility framework.

PICOS Domain	Eligibility Definition
**Population**	Adults aged ≥60 years with explicitly defined sarcopenia, frailty, prefrailty, or cognitive frailty. The clinical categories should be reported separately.
**Intervention**	Tai Chi, Baduanjin, Yijinjing, or another prespecified traditional Chinese mind–body exercise, either alone or as an adjunct.
**Comparator**	Usual care or no intervention, active exercise, or the same co-intervention received by the experimental group.
**Outcomes**	Physical performance and prespecified secondary outcomes. The availability of analyzable numerical data determined inclusion in a particular meta-analysis but did not determine eligibility for the systematic review.
**Study design**	Individually randomized or cluster-randomized controlled trials.

Note: PICOS, Population, Intervention, Comparator, Outcomes, and Study design.

**Table 2:** Basic characteristics of the included studies.

Study	Year	Disease	Diagnostic Criteria	Blinding Method	Intervention		Sample Size (*n*)		Age (Years)		Sex (M/F)		Outcome Measures	Intervention Duration
					EG	CG	EG	CG	EG	CG	EG	CG		
Wolf et al. [12]	2006	Frailty	Speechley–Tinetti classification	Single-blind (reported)	Training in six of the 24 simplified Tai Chi forms	Wellness education	158	153	81.00 ± 6.40	80.80 ± 5.90	10/148	10/143	Gait speed, 30CST	48 weeks
Dechamps et al. [13]	2009	Frailty	NR	NR	Yang-style 24-form Tai Chi training program	Cognition–action exercise program	26	26	80.80 ± 8.70	80.60 ± 9.20	8/18	14/12	MMSE, FOF, depressive symptoms, QOL, TUGT	24 weeks
Zhu et al. [14]	2017	Sarcopenia	AWGS (2014)	NR	Yijinjing exercise	No-intervention control	32	31	65.60 ± 11.40	66.30 ± 10.80	17/15	15/16	30CST, SST, grip strength	12 weeks
Zhu et al. [15]	2019	Sarcopenia	AWGS (2014)	NR	Simplified eight-style Tai Chi	Usual care	24	27	88.80 ± 3.70	87.50 ± 3.00	24/0	27/0	Muscle mass, grip strength, TUGT, gait speed, SST	8 weeks
He et al. [16]	2020	Frailty	TFI	NR	Baduanjin and horizontal foot exercises	Conventional treatment	30	30	69.58 ± 8.40	69.65 ± 7.92	21/9	20/10	SPPB, MBI	2 weeks
Kasim and Veldhuijzen van Zanten [17]	2020	Frailty	No formal diagnostic threshold; Fried frailty phenotype markers assessed	NR	Yang-style Tai Chi training	Zumba Gold	11	10	71.00 ± 3.10	70.00 ± 3.60	4/7	1/9	TUGT, 30CST, QOL, depressive symptoms	12 weeks
Zhou et al. [18]	2020	Sarcopenia	AWGS (2014)	NR	Baduanjin	Health education	20	20	72.67 ± 9.56	73.25 ± 8.50	8/12	9/11	Grip strength, 30CST, TUGT, FOF, BBS	8 weeks
Ge et al. [19]	2021	Prefrailty	Fried frailty phenotype	NR	Yang-style 24-form Tai Chi training program	Usual care	32	33	70.16 ± 5.40	72.91 ± 6.60	21/11	16/17	30CST, FOF, depressive symptoms, gait speed	8 weeks
Morawin et al. [20]	2021	Sarcopenia	No formal diagnostic threshold	NR	Yang-style 24-form Tai Chi training program	Health education	23	21	70.50 ± 5.80	70.50 ± 5.80	NR	NR	Muscle mass, gait speed, grip strength, 6MWT	10 months
Cheung et al. [21]	2022	Frailty	Fried frailty criteria	NR	Baduanjin Qigong	Light flexibility exercise	15	13	69.20 ± 3.88	69.46 ± 2.66	7/8	4/9	QOL, SPPB, depressive symptoms, MBI	8 weeks
Jiayuan et al. [22]	2022	Frailty	Fried phenotype + CDR = 0.5	Single-blind (reported)	Mindfulness-based Tai Chi Chuan	Mindfulness practices	30	30	71.30 ± 5.00	70.80 ± 4.20	14/16	13/17	SPPB, TUGT, 30CST, MMSE	6 months
Li et al. [23]	2022	Frailty	Fried frailty phenotype	NR	Tai Chi and resistance training	Usual care	30	27	82.87 ± 2.41	83.01 ± 2.82	10/20	11/16	Grip strength	30 weeks
Liu et al. [24]	2022	Frailty	Fried phenotype (1–2 criteria, as reported)	Single-blind (reported)	Tai Chi training	Usual activities	67	68	80.75 ± 2.99	80.74 ± 2.82	16/51	24/44	MMSE, QOL	12 months
Meng et al. [25]	2022	Frailty	Fried phenotype (3–5 criteria)	Double-blind (reported)	Eight-form Tai Chi and combined strength and endurance training	Combined strength and endurance training	50	50	76.31 ± 2.07	76.59 ± 2.58	20/30	21/29	Gait speed, grip strength, 6MWT, TUGT	24 weeks
Peng et al. [26]	2022	Sarcopenia	AWGS	NR	New Yijinjing exercise	Exercise therapy	40	40	72.12 ± 6.47	71.85 ± 5.73	17/23	16/24	Gait speed, BBS	8 weeks
Wang et al. [27]	2022	Frailty	Fried frailty phenotype	Double-blind (reported)	Baduanjin and combined strength and endurance training	Combined strength and endurance training	57	57	70.65 ± 3.73	70.74 ± 3.52	27/30	25/32	Gait speed, grip strength, 6MWT, TUGT	24 weeks
Wei et al. [28]	2022	Sarcopenia	AWGS (2016)	Double-blind (reported)	Yijinjing and resistance training	Resistance training	30	30	66.70 ± 4.10	66.87 ± 3.84	14/16	13/17	Muscle mass, grip strength	24 weeks
Xu et al. [29]	2022	Sarcopenia	NR	NR	Baduanjin and resistance training	Usual care	30	30	70.63 ± 0.85	70.73 ± 0.76	15/15	13/17	Gait speed, grip strength, SST, MBI	12 weeks
Chen et al. [30]	2023	Frailty	Chinese TFI	NR	Baduanjin and Zusanli acupressure	Conventional treatment and health education	20	22	85.20 ± 3.00	85.30 ± 2.40	9/11	12/10	Grip strength, depressive symptoms, QOL	12 weeks
Huang et al. [31]	2023	Sarcopenia	AWGS (2014)	NR	Baduanjin and health education	Health education	17	18	72.59 ± 4.27	71.50 ± 3.37	7/10	10/8	TUGT, SST	12 weeks
Peng et al. [32]	2024	Sarcopenia	NR	NR	Baduanjin and blood flow restriction training	Conventional treatment	63	63	70.65 ± 5.78	70.88 ± 6.02	28/35	30/33	Muscle mass, grip strength, MBI, SPPB	12 weeks
Tou et al. [33]	2024	Frailty	Fried phenotype; AWGS 2019 low-HGS inclusion	Assessor-blind	Community-based Baduanjin Qigong	Wait-list control	29	27	72.90 ± 8.00	72.60 ± 5.70	5/24	2/25	Gait speed, grip strength, 30CST, TUGT, FOF, depressive symptoms	16 weeks

Note: 6MWT, 6 min walk test; 30CST, 30 s chair stand test; AWGS, Asian Working Group for Sarcopenia; BBS, Berg Balance Scale; CDR, Clinical Dementia Rating; CG, control group; EG, experimental group; F, female; FOF, fear of falling; HGS, handgrip strength; M, male; MBI, Modified Barthel Index; MMSE, Mini-Mental State Examination; *n*, number of participants; NR, not reported; QOL, quality of life; SPPB, Short Physical Performance Battery; SST, sit-to-stand test; TFI, Tilburg Frailty Indicator; TUGT, Timed Up and Go Test.

## Data Availability

No new data were created or analyzed in this study. Data sharing is not applicable to this article.

## Supplementary Materials

The following supporting information can be downloaded at: https://www.mdpi.com/article/10.3390/healthcare14162664/s1, Table S1: PRISMA 2020 Checklist [65].

## Appendix A

**Table A1:** GRADE Summary of Findings framework.

Critical Outcome	Evidence	Reported Effect (95% CI)	Risk of Bias	Inconsistency	Indirectness	Imprecision	Publication Bias	Certainty
Gait speed	9 studies	MD 0.06 m/s (−0.07 to 0.18)	Serious	Very serious	Not serious	Serious	Undetected	⨁◯◯◯ Very low
TUGT	9 studies	MD −1.17 s (−1.80 to −0.54)	Serious	Serious	Not serious	Not serious	Undetected	⨁⨁◯◯ Low
SPPB	4 studies	MD 0.99 points (0.58 to 1.40)	Serious	Not serious	Not serious	Serious	Undetected	⨁⨁◯◯ Low
Grip strength	12 studies	MD 2.02 kg (0.58 to 3.47)	Serious	Serious	Not serious	Not serious	Undetected	⨁⨁◯◯ Low
Quality of life	5 studies	SMD 0.61 (−0.10 to 1.33)	Serious	Serious	Not serious	Serious	Undetected	⨁◯◯◯ Very low

Certainty of evidence was assessed across risk of bias, inconsistency, indirectness, imprecision, and publication bias. CI, confidence interval; GRADE, Grading of Recommendations Assessment, Development and Evaluation; MD, mean difference; SMD, standardized mean difference; TUGT, Timed Up and Go Test; SPPB, Short Physical Performance Battery. One filled symbol denotes very low certainty, and two filled symbols denote low certainty.

**Table A2:** Exploratory univariable meta-regression analyses for grip strength.

Moderator	No. of Studies	Coefficient, kg	SE	95% CI	*p* Value
Diagnostic group: sarcopenia versus frailty	12	0.090	1.772	−3.858 to 4.039	0.960
Intervention duration, per week	12	−0.023	0.085	−0.211 to 0.166	0.793

Coefficients are reported with standard errors and 95% confidence intervals. CI, confidence interval; SE, standard error.
